# Detectable quorum signaling molecule via PANI-metal oxides nanocomposites sensors

**DOI:** 10.1038/s41598-024-60093-8

**Published:** 2024-05-02

**Authors:** Walaa S. Gado, Abdalrahman G. Al-Gamal, Mona Shaban E. M. Badawy, A. Labena, Khaled Zakaria, Khalid I. Kabel

**Affiliations:** 1 Egyptian Petroleum Research Institute (EPRI), 11727, Nasr City, Cairo Egypt; 2 Department of Microbiology and Immunology, Faculty of Pharmacy (Girls), Al-Azhar University, Cairo, Egypt

**Keywords:** Quorum sensing, Sensors, Conducting polymers, Metal oxides, Nanocomposites, AHL, SRB-Biofilm, Conjugated polymers, Characterization and analytical techniques, Pollution remediation

## Abstract

The detection of N-hexanoyl-l-homoserine lactone (C_6_-HSL), a crucial signal in Gram-negative bacterial communication, is essential for addressing microbiologically influenced corrosion (MIC) induced by sulfate-reducing bacteria (SRB) in oil and gas industries. Metal oxides (MOx) intercalated into conducting polymers (CPs) offer a promising sensing approach due to their effective detection of biological molecules such as C_6_-HSL. In this study, we synthesized and characterized two MOx/polyaniline-dodecyl benzene sulfonic acid (PANI-DBSA) nanocomposites, namely ZnO/PANI-DBSA and Fe_2_O_3_/PANI-DBSA. These nanocomposites were applied with 1% by-weight carbon paste over a carbon working electrode (WE) for qualitative and quantitative detection of C_6_-HSL through electrochemical analysis. The electrochemical impedance spectroscopy (EIS) confirmed the composites’ capability to monitor C_6_-HSL produced by SRB-biofilm, with detection limits of 624 ppm for ZnO/PANI-DBSA and 441 ppm for Fe_2_O_3_/PANI-DBSA. Furthermore, calorimetric measurements validated the presence of SRB-biofilm, supporting the EIS analysis. The utilization of these MOx/CP nanocomposites offers a practical approach for detecting C6-HSL and monitoring SRB-biofilm formation, aiding in MIC management in oil and gas wells. The ZnO/PANI-DBSA-based sensor exhibited higher sensitivity towards C_6_-HSL compared to Fe_2_O_3_/PANI-DBSA, indicating its potential for enhanced detection capabilities in this context. Stability tests revealed ZnO/PANI-DBSA's superior stability over Fe_2_O_3_/PANI-DBSA, with both sensors retaining approximately 85–90% of their initial current after 1 month, demonstrating remarkable reproducibility and durability.

## Introduction

Bacteria communicate with each other in their surrounding medium through the generation of chemical signaling molecules, chemotactic particles, or a pheromone known as quorum sensing (QS) molecules. It has been reported that there is a direct correlation between the chemical signaling molecules and bacterial concentration^1,2^. In addition, bacteria use QS not only to communicate but also to regulate/synchronize the expression of numerous genes that are necessary for bacteria to function as a community^3,4^. Biofilms are microbial communities characterized by bacterial cells that are attached to a substrate. These cells, when attached to a substrate, aggregate within a matrix of extracellular polymeric substances (EPS) that produce and exhibit variable phenotypes that influence the growth rate and gene replication^5,6^. Key factors such as nutrient availability^7^, chemotaxis in the surface direction^8^, bacterial movement^9^, attachment to the surface, and surfactant existence^10^ are some impacts that affect the formation of the biofilm. Typically, Gram-negative bacteria generate a chemical signal molecule known as N-acylated homoserine lactones (AHLs). Recently, researchers discovered a valuable pathway to monitor bacterial activities and colony settlement size through the assessment of AHLs^11–14^. Sulfate-reducing bacteria (SRB), as a group of Gram-negative bacteria, have been recognized as one of the most harmful bacteria for iron metals utilized in oil and gas fields^14,15^. SRB induces microbially influenced corrosion (MIC) for iron metals by producing corrosive metabolites such as hydrogen sulfide gas (H_2_S), assumed electrochemical (cathodic depolarization), and microbial colonization (biofilm formation)^15^. SRB produces many AHLs, including N-hexanoyl homoserine lactone (C_6_-HSL) and N-dodecanoyl homoserine lactone (C_12_-HSL)^14^. Many efforts have been made to detect SRB-AHL signals to detect and inhibit such microbial communities. Many conventional techniques such as HPLC-MS/MS^16^, HPLC/ESI-MS^17^, LC-MS^18^, GC–MS^19^, and other chromatographic examinations are considered inadequate techniques due to their time consumption and high cost^11,12^. It has been noted that few researchers were focused on detecting SRB-biofilms using smart devices like sensors. In the last decade, a simple non-destructive technique like electrochemical impedance spectroscopy (EIS) was employed for this purpose^20^. EIS has been employed to detect AHLs using a working electrode (WE) produced by a screen-printed electrode (SPE) technique^12^. The WE is fabricated based on metals^21^, metal oxides (MO_x_)^11,12^, or metal/ metal oxide combination^22^ which can be utilized alone or embedded in a polymer matrix and/or one of the carbon forms materials. Research studies confirmed this method's accuracy, rapid response, and sensitivity for estimating the chemical signals resulting from bacterial assemblies compared with other electrochemical methods^12,13,23^. MO_x_ including Zinc oxide (ZnO)^24^, and Ferric oxide (Fe_2_O_3_)^1^ displayed good performance in sensing AHLs with high sensitivity, fast response, and ease of integration into compact electronic devices like SPE. However, MOx-based sensors suffer from low selectivity^25^ and conflicting measurements with other dissolved ions in formation water, as well as being operated under a high-temperature process (>100 °C)^26^. Conductive polymers (CPs) are considered successful alternative materials to MOx as sensitizers of organic molecules^27,28^. Indeed, CPs present the advantages of easy preparation through chemical or electrochemical procedures, and they can sense selective ions in various media (basic, acidic, and neutral) at room temperature^29^. Nevertheless, their long-term stability due to a moisture uptake need to be improved^30–33^. CPs exhibit distinctive properties that find valuable applications in sensor design. Consequently, conducting polymers based on Molecularly Imprinted Polymers (MIPs), such as polypyrrole, polythiophene, poly(3,4-ethylenedioxythiophene), polyaniline, and ortho-phenylenediamine, are commonly employed in sensor development^34^. Conducting polyaniline (PANI) showed great potential in the field of electrochemical applications, including sensors, biosensors, and supercapacitors. This can be attributed to its superlative characteristics, including affordability, strong stability, electrical conductivity, and widespread availability^35–38^.

In the realm of biosensor design, the selection and integration of advanced materials play a pivotal role in enhancing sensitivity, selectivity, and overall performance^39^. Among the array of materials employed in biosensor development, ZnO^40,41^, Fe_3_O_4_, polyaniline (PANI), and dodecylbenzene sulfonic acid (DBSA) have emerged as noteworthy candidates, each contributing unique characteristics to the biosensing landscape^42^. ZnO, a versatile semiconductor, exhibits exceptional properties such as high surface area, biocompatibility, and ease of functionalization. These attributes make ZnO a compelling choice for biosensor applications, where precise and efficient detection is paramount^40,41,43^. Fe_2_O_3_ nanoparticles are experiencing a surge of interest within the realm of biosensing applications owing to their advantageous band gap, biocompatibility, lack of toxicity, thermal stability, intriguing optical and magnetic characteristics, and abundant natural presence^44,45^. These nanoparticles hold promising potential in electrochemical sensing due to their remarkable electrical conductivity, even under ambient conditions, facilitated by electron exchange between Fe^2+^ and Fe^3+^ ions^46^. Nonetheless, traditional metal oxides typically necessitate elevated temperatures for optimal sensing performance. To circumvent these challenges, the exploration oforganic–inorganic hybrid nanocomposites, amalgamating metal oxides with conducting polymers, presents a compelling avenue for the commercial advancement of cost-effective electrochemical sensors^47^. PANI, a conducting polymer, offers a distinct advantage with its tunable conductivity and redox properties. Its facile synthesis and ability to undergo reversible doping and de-doping processes make PANI an attractive material for biosensing platforms, allowing for tailored responses to specific analytes^48^. Dodecylbenzene sulfonic acid, serving as a surfactant and dopant^42^, contributes to the stabilization and enhancement of the electrical properties of conducting polymers like PANI. Its role in promoting uniform film formation and facilitating electron transfer processes further solidifies its significance in biosensor design^49^.

The interaction mechanism between these materials involves the formation of a composite structure where ZnO or Fe_2_O_3_ nanoparticles are coated with a layer of PANI, facilitated and stabilized by DBSA. This composite structure not only provides a conducive and stable platform for biomolecule immobilization but also enhances the overall performance of the biosensor in terms of sensitivity, specificity, and response time^50,51^.

In this article, promising nanomaterials with a high sensitivity to C_6_-HSL molecules were prepared depending on divalent nanometal oxides represented in ZnO and Fe_2_O_3_ doped in CPs matrix, polyaniline-dodecylbenzene sulfonic acid (PANI-DBSA). Afterward, the synthesized composites were confirmed structurally and surface morphologically using several analyses including FT-IR, Raman, XRD, SEM, HR-TEM, GPC, and DLS. The fabricated sensors were evaluated under the environmental conditions of oil wells infected with SRB by the EIS. In addition, the SRB-biofilm was detected by evaluating the corrosion rate (C_R_) of the carbon steel at ambient conditions and the results were confirmed with compatible colorimetric analysis.

## Experimental work

### Synthesis of MO_x_ NPs and PANI-DBSA/MO_x_ nanocomposites

ZnO NPs^52^ and Fe_2_O_3_^53^ were synthesized by the co-precipitation method according to the corresponding references, more details are illustrated in the supplementary materials (SM). The PANI-DBSA nanocomposites with MO_x_ were prepared by an emulsion polymerization technique. Briefly, 10 mmol of the DBSA was prepared by diluting the concentrated acid with distilled water in a ratio of 1: 9, respectively. After that, 0.3 moles (30 wt %) of the MO_x_ (ZnO or Fe_2_O_3_) were added^54^, and then the suspensions were mechanically stirred to obtain a homogeneous distribution of the MO_x_ in the DBSA solution. Afterwards, the molecular equivalent by weight of aniline monomers was added followed by a satisfied stirring to get the aniline-DBSA salt. The aniline-DBSA salt was polymerized immediately after slowly adding 5 mmol of KPS to the emulsion at 0 °C. The solution was kept at 0 °C to complete the polymerization process. Finally, the prepared composites were washed with methanol and distilled water to eliminate any contamination, then dried in an oven at 50 °C for 3 h to obtain ZnO/PANI-DBSA, Fe_2_O_3_/PANI-DBSA nanocomposites. The non-doped PANI-DBSA was also prepared according to the same procedure mentioned before except for the MO_x_ NPs addition step.

### Electrochemical sensing of enriched-SRB biofilm and C_6_-HSL

#### SRB media preparation and cultivation

The bacteria consortium in this study was obtained from a water sample with a salinity content (NaCl) of 2.6% that was collected from a water tank of North Bahria, Qarun Petroleum Company, western desert, Egypt. In the field, the water sample was enriched using an anaerobic selective media (modified Postgate´s-B medium) according to Postgate^55^. Modification in Postgate’s medium composition was done using the original water salinity (NaCl) of 2.6% and pH of 6.76 during media preparation. Media preparation, enrichment, and cultivation were according to the modified Hungate's technique for anaerobes^56^. Afterward, the enriched SRB sample was further enriched and used as inocula for the cultivated reactors at 37 °C for 14 days and the black precipitation (Ferrous sulfide) was noticed^55^. The bacterial count was estimated for the inocula using the most probable number (MPN)^57^. A mild steel coupon AISI 1018 (mild/low carbon steel strip COSASCO's, Rohrback Cosasco Systems, Inc) with a dimension of 1.0 × 1.0 × 0.32 cm was used as a working electrode. The chemical composition of the coupons was carbon, C 0.14–0.20%, Iron, Fe 98.81–99.26% (as remainder), Manganese, Mn 0.60–0.90%, Phosphorous, P ≤ 0.040% and Sulfur, S ≤ 0.050%. The mild steel coupons were polished with a series of emery paper with different grades (320, 400, 600, 800, 1000, 1200), followed by ethanol degreasing. The sample was characterized by its microbial community using 16S metagenomics sequencing (data not shown). In addition, the bacterial community’s corrosive activities were evaluated by measuring dissolved sulfide according to the “German Standard Methods using DR3900 (Hach Lange GmbH, Berlin, Germany)” and the change in redox potential, using “SenTix ORP electrode, WTW” was evaluated. Moreover, metal corrosion rate was also measured using weight lost method, open circuit potential (OCP) and the electrochemical impedance spectroscopy (EIS) (date not shown). In order to visualize the bacterial biofilm on the metal surface scanning electron microscopy (SEM was used. The phylogenetic analysis showed that there are one dominant taxon affiliated to family Desulfovibrionales. This sequence was represented by genera of Desulfovibrio (data not provided).

#### Assessment of enriched-SRB biofilm formation

In a conventional glass three-electrodes cell, the electrochemical measurements of open circuit potential (OCP) and electrochemical impedance spectroscopy (EIS) were tested by Origa Flex potentiostat–galvanostat system under anaerobiotic circumstances at an ambient temperature. The culture media, previously described, was used for all studies, either inoculated or not with the enriched SRB. The OCP was accomplished by allowing the WE to soak in the solution under investigation for 30 min before conducting the electrochemical tests.

#### Assessment of C_6_-HSL

The obtained composites: ZnO/PANI-DSBA and Fe_2_O_3_/PANI-DSBA were mixed with the carbon paste (CC) with a percentage not exceeding 1% and deposited as sensing materials for C_6_-HSL on the working electrode of the SPE sensor as shown in Fig. S1a and b. The prepared sensors were evaluated using the EIS technique for measuring the bacterial signals by immersing them in customized anaerobic cells inoculated by enriched SRB with different C_6_-HSL concentrations. The SRB bacteria were allowed to grow in the incubator and then the SRB media were transferred to the measurement cell containing the sensor. The main target of the sensor that has the active materials (PANI-DBSA/ZnO or PANI-DBSA/Fe_2_O_3_) on the top of the working electrode reacts with the signal (C_6_-HSL) produced by the SRB bacteria assembly and detects its concentration as a function of changing the of EIS performance. The cyclic voltammetry (CV) carried out the selected potential window between 0.3 V and 0.8 V. Before testing the fabricated sensors using CV the sensor was dried in the oven for 30 min at 60 °C under N_2_ followed by blowing it with vacuumed air to remove any attached contaminations. The EIS measurements were fulfilled by the Origalys–signal multi-channel system (OrigaFlex 01A) with a frequency range of 0.01Hz-100 kHz with an amplitude of 150 mV. The recorded Nyquist plot of the impedance data was fitted by Zsimwin Version 3.0.1 software, see Fig. S2.

#### Colorimetric detection of C_6_-HSL

The biosensor Agrobacterium tumefaciens KYC55 (pJZ372; pJZ384; pJZ410) was used for the detection of the C_6_-HSL production. This bacterial strain is responsible for the expression of a lacZ fusion in response to the C_6_-HSL. This leads to cleavage of 5-Bromo-4-chloro-3indolylgalactopyranoside (X-Gal) (Promega, Madison, WI, USA) and staining the agar plates with blue color as an indication for the C_6_-HSL and recorded as a positive result^58^. The C_6_-HSL production was assessed as (+ 1) for the positive medium production of the C_6_-HSL in comparison to the positive (+ ve) and negative (-ve) controls^58^. Further information describing the procedure of the colorimetric detection of the C_6_-HSL using the biosensor colorimetric technique was demonstrated in the supplementary materials.

## Results and discussion

### Characterizations of the prepared PANI-DBSA/MO_x_ nanocomposites

**Figure 1:**
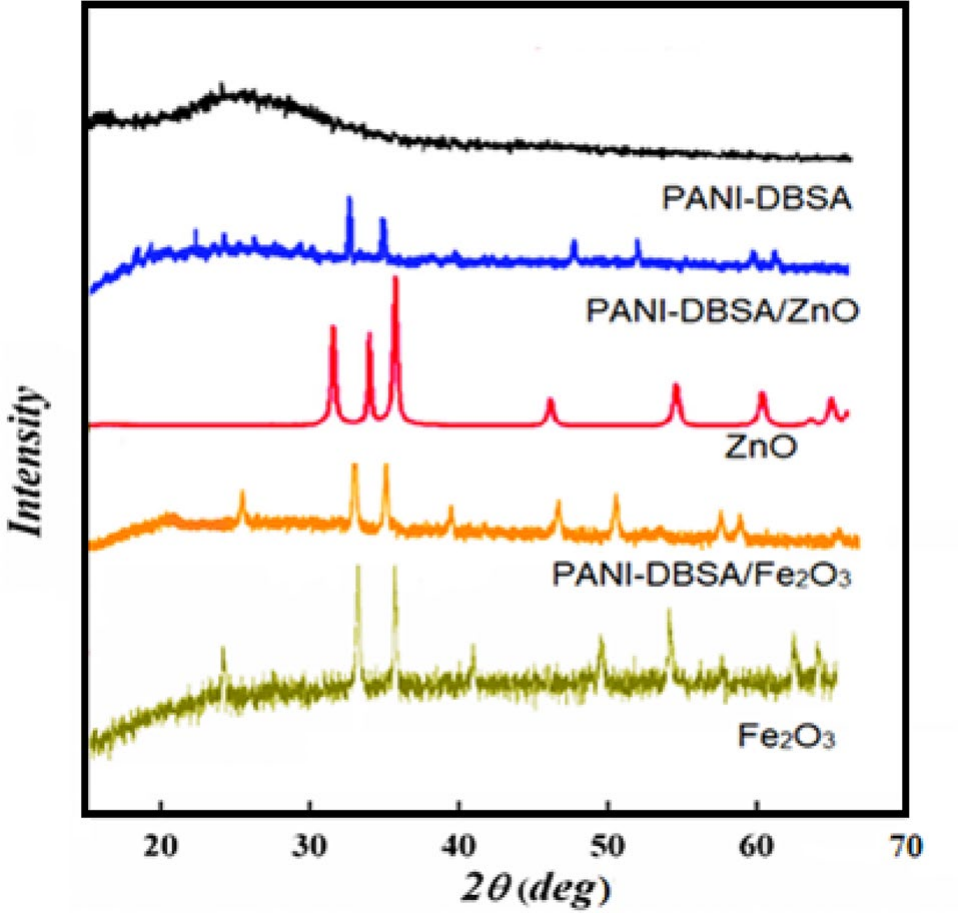
XRD patterns of the PANI-DBSA, ZnO/PANI-DBSA, ZnO, Fe_2_ O_3_ /PANI-DBSA, and Fe_2_O_3_.

The XRD of the prepared PANI-DBSA exhibits broad diffraction peaks from 10° to 30° because of vertical and parallel periodic arrangement corresponding to the PANI chains, this displayed its amorphous structure via two peaks at 2θ = 20 and 25°. The (110) plane was indexed for this reflection (Fig. 1).

The XRD patterns of the prepared materials are displayed in Fig. 1. The particles' average sizes were calculated by Debye–Scherrer’s equation as follows^59,60^:

1 $${\text{D}}=\frac{0.9\lambda }{\beta cos\theta }$$

where D is the crystal size, λ is the x-ray wavelength, β is the full width at half maximum of the diffraction peak, and θ is the Bragg diffraction angle.

The data demonstrated that; all the peaks’ values can be referred to as the hexagonal structure of ZnO NPs, distinct diffraction peaks at (100), (002), (101), (102), (110), (103), (200), (112), and (201) in the pattern matched with those in the JCPDS card (Card No. 89-1397). The high intensity and the narrow width of the ZnO diffraction peaks confirmed the resulting product as it has a high crystallinity with a calculated average size of 39.95 nm. The XRD pattern of the PANI-DBSA matrix incorporating ZnO affirms the creation of nanocomposites. The presence of ZnO induces changes in the amorphous nature of PANI, evidenced by characteristic peaks arising from hydrogen bond formation between the H–N of PANI and the oxygen of ZnO nanoparticles at 25°. Scherrer's equation calculates the crystalline size, D, of the ZnO/PANI-DBSA nanocomposite as 86 nm, indicating the impact of ZnO nanoparticle interaction on the composite's structural characteristics.

Moreover, the distinctive diffraction peaks of Fe_2_O_3_ at 2θ = 23.89°, 34.37°, 37.22°, 41.01°, 48.99°, 55.22°, 63.12°, and 65.21°, correspond to crystal planes (0 1 2), (1 0 4), (1 1 0), (1 1 3), (0 2 4), (1 1 6), (2 1 4), and (3 0 0), respectively. All peaks were well matched with those in the JCPDS card for the Fe_2_O_3_ (ICDD card no. 330664. Utilizing Scherrer's formula, the average crystallite size is determined to be 43.23 nm. The XRD pattern of the PANI matrix incorporating Fe_2_O_3_ shows that the prominent polyaniline peak at 25° appears gradually, suggesting the incorporation of iron oxide into the PANI-DBSA matrix. Simultaneously, the characteristic peaks of iron oxides also undergo a corresponding attenuation. By applying Scherrer's equation, the crystalline size of the Fe_2_O_3_ /PANI-DBSA nanocomposite refers to 78 nm, indicating the impact of Fe_2_O_3_ nanoparticle interaction on the composite's structural characteristics.

Thus, the incorporation of ZnO or Fe_2_O_3_ nanoparticles into the PANI-DBSA composite leads to lattice parameter changes which can affect the diffraction angles, causing peak shifts, and might induce strain in the composite material, leading to peak shifts. This strain can arise from lattice mismatches between the PANI matrix and the nanoparticles. Also, this interaction between the PANI and the nanoparticles may induce changes in the crystalline structure of either the PANI or the nanoparticles.

**Figure 2:**
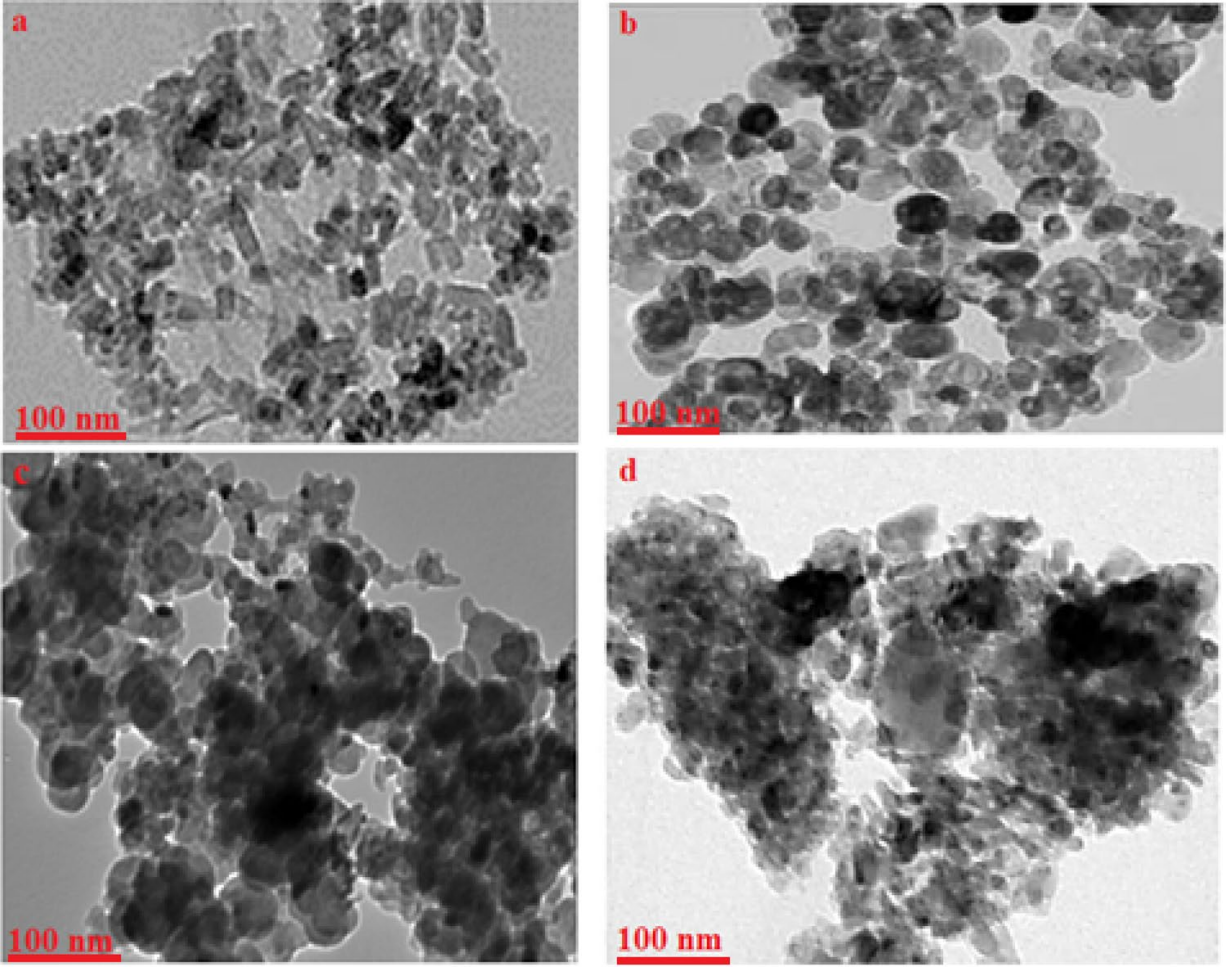
HR-TEM images of (**a**) ZnO NPs, (**b**) Fe_2_O_3_ NPs, (**c**) ZnO/PANI-DBSA, and (**d**) Fe_2_O_3_/PANI-DBSA.

The HR-TEM image (see Fig. 2a) provided visual evidence of the nanocrystalline particles which consisted of hexagonal and rod-like morphologies of ZnO NPs. The images revealed a slight agglomeration of these particles. In addition, the HR-TEM images, as depicted in Fig. 2b, provided additional confirmation of the spherical shape of the Fe_2_O_3_ nanoparticles which exhibited a slight agglomeration. The diameters of the nanoparticles range from 50 to 65 nm, and these estimated values closely align with those of XRD. This result confirmed the accuracy of the size determination based on the TEM analysis and supported the understanding of the Fe_2_O_3_ nanoparticle characteristics. Furthermore, the TEM image (see Fig. 2c) of the nanocomposite featuring ZnO nanostructures revealed the dispersion of the spherical ZnO nanoparticles with sizes ranging from 35 to 45 nm throughout the polymer matrix. Also, Fig. 2d provided the morphology of the Fe_3_O_4_/PANI-DBSA as an aggregate of very fine particles with uniform structure. Furthermore, it completely differs from the pure Fe_3_O_4_ particles (Fig. 2c) which exhibited a spherical shape that undergoes significant changes upon the PANI-DBSA doping. The presence of dark spots in the TEM image corresponded to these nanoparticles and indicated their presence within the composite material.

The molecular weight of the prepared PANI-DBSA was measured via GPC analysis. The average molecular weight (M_w_) was 15,080 g/mol and the polydispersity (PDI) was 3.6. Moreover, the structure confirmation and morphological characteristics of the PANI-DSBA were investigated using FT-IR (Fig. S3) and SEM (Fig. S4), respectively. The size distributions of the prepared MOx particles were determined by DLS (Fig. S5). Additionally, Raman's analysis of all the prepared materials is presented in Fig. S6.

### Electrochemical assessment of enriched-SRB biofilm and C_6_-HSL

#### Electrochemical Assessment of enriched-SRB biofilm using SPE

The MIC behavior of the carbon steel specimens immersed in an anaerobic modified Postgate´s-C media (sterile uninoculated and enriched SRB-containing media) was evaluated using OCP, which is discussed in SM (see Fig. S7), and EIS to inspect the influence of SRB-biofilm on the carbon steel. The measurements were investigated after 5 days of biofilm formation intervals for 30 days under static and anaerobic conditions.

##### EIS data analysis

**Figure 3:**
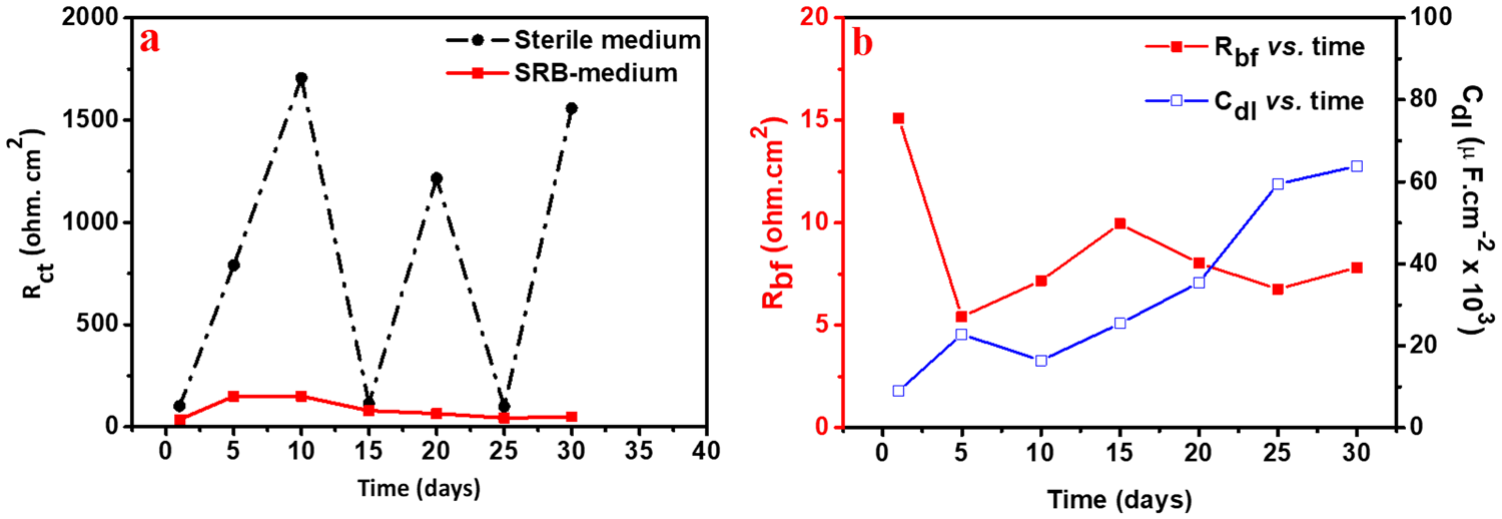
(**a**) The variation of charge transfer resistance (R_ct_) vs. immersion time for both bio-medium. (**b**) The difference in the bio-film resistance (R_bf_) and electrical double layer capacitance (C_dl_) vs. immersion time for carbon steel for the SRB system.

Figures S8a and b displayed the Nyquist curves with time for the carbon steel immersed in the control and enriched-SRB media under anaerobic conditions favorable for bacterial growth, respectively. To explain the experimental EIS spectra acquired in the case of a biofilm formed on the carbon steel surface, the acquired data were fitted and simulated with two suitable electrical equivalent circuit models Rs (Q_dl_ R_ct_) and Rs (C_bf_ (R_bf_ (C_dl_ R_ct_)) for the sterile and enriched-SRB media, respectively as illustrated in Fig. S9, where Rs denotes solution resistance, Q_dl_ represented constant phase element (CPE), double-layer capacitance (C_dl_), R_bf_ denotes biofilm resistance, and R_ct_ signifies charge transfer resistance. The fitting EIS data are listed in Tables 1, 2. Figure S8 displayed that; by increasing the immersion time for the exposed metal coupons to the various media, the diameter of the Nyquist curves shifts a lot. In the presence of the enriched-SRB (Fig. S8b), after 20 days of immersion, the diameter of the Nyquist curves reached its highest value which indicates that a protective coating made of the EPS and corrosion products has assembled on the surface of the carbon metal steel. Alternatively, the diameter of the Nyquist plots gradually decreased at 25 and 30 days of immersion times. It is worth mentioning that the Nyquist plot diameter amplified as a result of the biofilm-corrosion formation which offers more metal protection, while the diminishing behavior, indicated the presence of a permeable or discontinuous coating which stimulates the disintegration of the SRB-biofilm and the protective passive layer^61^. Inspection of Tables 1 and 2, it was noted that both media have good conductivity due to the solution resistance (Rs) being overly low and changing slightly with disclosure time, where the values of the Rs were significantly smaller in the enriched SRB-medium than in the sterile medium after 5 days of immersion as a consequence of the producing of bacterial solubilized metabolites such as pyruvic acid^62^. From the electrochemical point of view, the size of the capacitive circle at a low frequencies region was represented by the diameter of the semicircle, pointing out the difference in the charge transfer resistance (R_ct_) that expresses the anodic reaction growth was ruled by the charge transport process. Interestingly, as outlined in Table 1, the R_ct_ values were observed to have a variability trend in the sterile medium which can be referred to the occurrence of chlorides ions from the oilfield water samples, causing pitting corrosion on the carbon steel surface (decreasing R_ct_, at periods of 1, 15 and 25 days being about 102.4, 114.7 & 99.22 Ω.cm^2^, respectively. On the contrary, at the immersion periods of 5, 10, 20, & 30 days, the R_ct_ values increased to 790.9, 1706, 1216, and 1559 Ω.cm^2^, respectively, confirming the metallic passivation as shown in Fig. S8a^63^. Further investigation (see Table 2) it is obvious that in the enriched SRB-medium, the increase of the R_ct_ value was attained at an immersion time of 10 days and has a value of 149.7 Ω.cm^2^, attributing to enriched-SRB biofilms on the metal surface and certain areas of the surface were covered by microbial clusters, leading to the surface resistance of the metal to increase^64^. After 15 days of immersion, there was a decrease in the R_ct_ value to 79.59 Ω.cm^2^ and then achieved a relatively stable state until 30 days, indicating an upsurge in charge transfer between the enriched-SRB biofilm and the electrode surface. With successive decreases in the R_ct_ values, the enriched-SRB amplified the corrosion rate of the carbon steel through the production of biofilm, formation of sulfide, and consequent accumulation of conductive iron sulfide layers with the extent the immersion period from 15 days up to the last day at 30 days. These porous and conductive FeS films formed on the metallic surface of the electrode enhanced the corrosion kinetics in the presence of SRB. Figure 3a depicts the variation of charge transfer resistance (R_ct_) with the immersion time. Regarding the bio-film resistance (R_bf_) values for SRB medium, it is apparent from Table 2, it is apparent that the R_bf_ value greatly decreases with immersion time, an embodiment that the charge transfers at the biofilm/the WE electrode surface interface become faster, which increases the MIC rate. Figure 3b presents a difference in the bio-electrochemistry behavior of the SRB system in terms of the biofilm resistance (R_bf_) and the C_dl_ with time. What is more, the R_bf_ values in the SRB medium significantly decreased in the first 5 days of immersion of carbon steel starting from 15.11 to 5.420 Ω.cm^2^, implying that the initially formed biofilm lost some of its structural parts which exposed the metal surface to the corrosive species existing in the solution and confirming the instability of this bio-film^65^. Concerning the C_dl_ values, the results show that they increased during the periods of immersion which assisted the presence of the bio-film porosity and accelerated the rate of carbon steel corrosion. Generally, the instability of the capacitance depends upon the formation of the biofilm at the interface and is related to the increase of active regions through the pores forming in the biofilm as a result of the microbiological growth phases.

**Table 1:** The important electrochemical impedance parameters for the carbon steel corrosion immersed in sterile medium.

Time (d)	Sterile medium			
	R_s_ (Ω cm^2^)	R_ct_ (Ω cm^2^)	Q_dl_	n
1	1.848	102.4	0.002593	0.7417
5	4.401	790.9	0.001032	0.8565
10	4.041	1706	0.001182	0.8626
15	3.490	114.7	0.001656	0.8312
20	2.913	1216	0.001519	0.8678
25	3.022	99.22	0.002009	0.8696
30	3.515	1559	0.001768	0.8764

**Table 2:** Electrochemical impedance parameters of the carbon steel corrosion immersed in SRB medium.

Time (d)	SRB medium				
	R_s_ (Ω cm^2^)	R_ct_ (Ω cm^2^)	R_bf_ (Ω cm^2^)	C_bf_ (F cm^−2^)	C_dl_ (F cm^−2^)
1	4.094	36.1	15.11	0.002883	0.008979
5	2.122	149.1	5.420	0.006763	0.02281
10	2.036	149.7	7.173	0.01914	0.0164
15	2.269	79.59	9.951	0.03845	0.02542
20	2.825	64.94	8.028	0.03266	0.03541
25	2.427	43.41	6.771	0.03878	0.05946
30	2.136	49.5	7.815	0.04295	0.06384

##### Electrochemical assessment of C_6_-HSL using SPE

**Figure 4:**
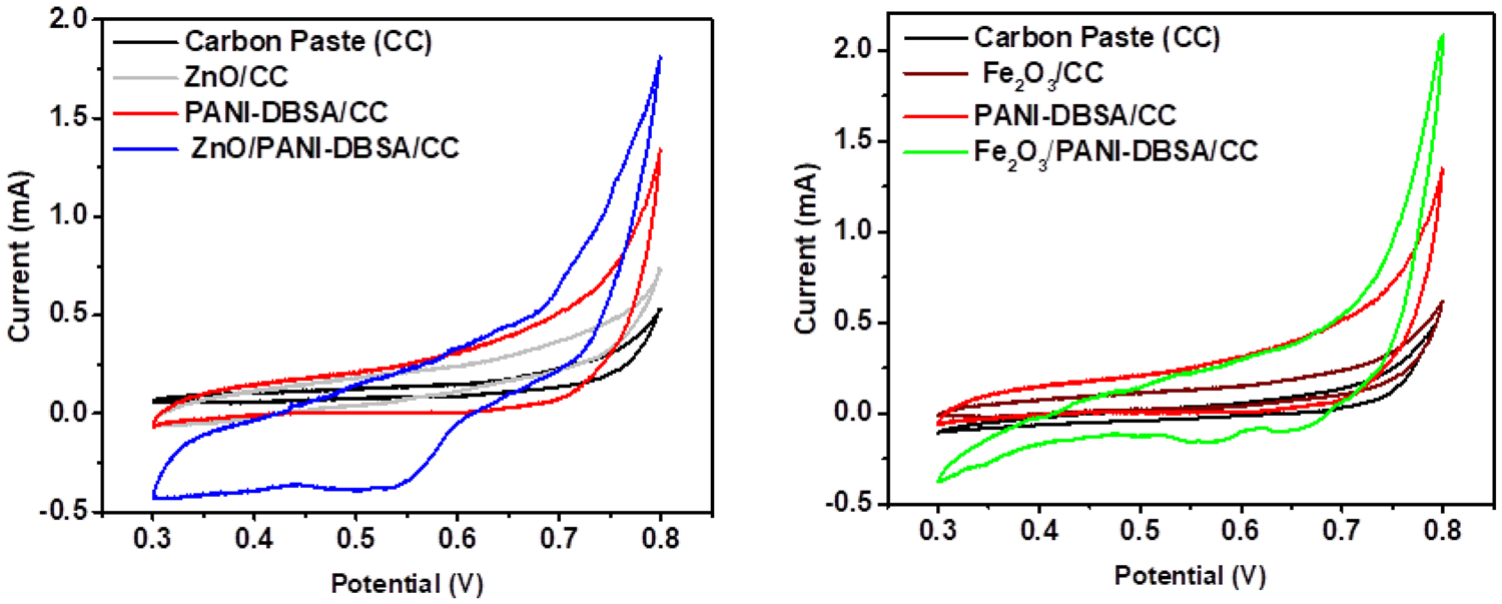
CV of carbon paste and altered electrodes based on carbon paste, MO_x_ (ZnO and Fe_2_O_3_) PANI-DBSA, PANI-DBSA/MOx composites (ZnO/PANI-DSBA and Fe_2_O_3_ /PANI-DSBA in 1000 ppm C_6_-HSL.

The electrochemical characteristics of the carbon paste and altered electrodes were examined using CV. A solution containing 1000 ppm C_6_-HSL to analyze the mass process of the adjusted electrode. As shown in Fig. 4, the cathodic peak current of both ZnO/PANI-DBSA and Fe_2_O_3_/PANI-DBSA-based WE surpassed that of the others, indicating superior electrical conductivity^66,67^. Also, it was observed that the ZnO/PANI-DBSA is more cathodic toward reactive than Fe_2_O_3_/PANI-DBSA this is attributed to the state true of that ZnO nanoparticles are more conductive than Fe_2_O_3_ at the same ratio in their composites. The conductivity of the ZnO nanoparticles arises from defects in oxygen vacancies, which create donor states within the bandgap^68,69^. In the case of the ZnO/PANI-DBSA-based sensor, CV displayed a reduction shifted from 0.704 V for PANI-DBSA-based sensor to 0.543 V peak while this peak moved slightly in the case of Fe_2_O_3_/PANI-DBSA based sensor to 0.645 V. Here, with the presence of the MO_x_ in the PANI-DBSA based sensors, the reduction peak current potential less positive which can be attributed to both ZnO and Fe_2_O_3_/PANI-DBSA based sensor stimuli the reduction of lactone ring of C_6_-HSL.

**Figure 5:**
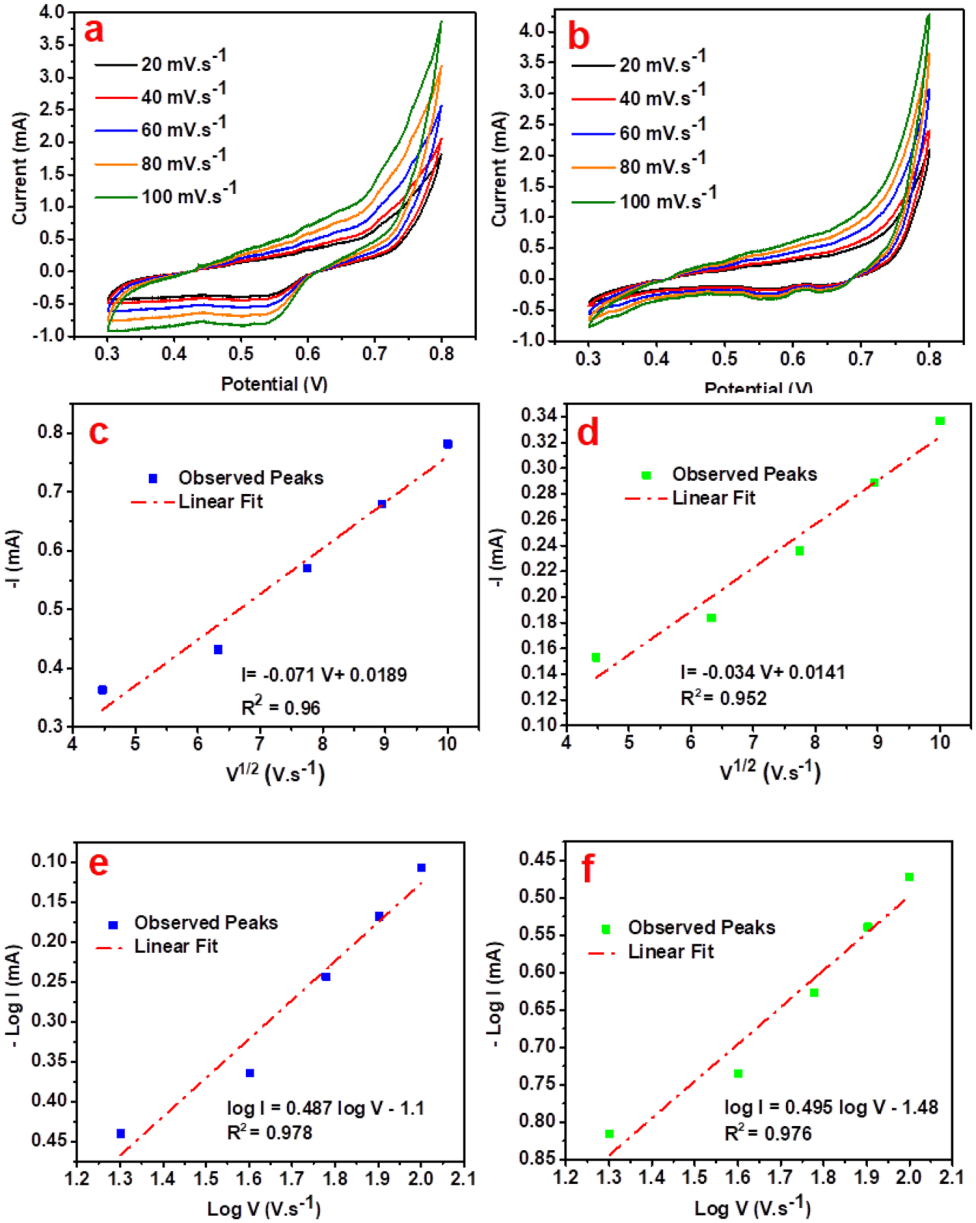
Cyclic voltammograms of C_6_-HSL (1000 ppm) at different scan rates (downtoup:20, 40, 60, 80, and 100 mVs^−1^) on (**a**) ZnO/PANI-DSBA/CC and (**b**) Fe_2_O_3_/PANI-DSBA/CC. Linear relationships of cathodic current peaks versus square root of the scan rate (ν^1/2^) of (**c**) ZnO/PANI-DSBA/CC and (**d**) Fe_2_O_3_/PANI-DSBA/CC working electrode against Ag/AgCl reference electrode. Plot of logarithm of the scan rate (log ν /mV s^−1^) vs. logarithm of current peaks (log I) of (e) ZnO/PANI-DSBA/CC and (**f**) Fe_2_O_3_/PANI-DSBA/CC.

The effect of scan rate on the two ZnO/PANI-DSBA and Fe_2_O_3_ /PANI-DSBA based sensors were examined as shown in Fig. 5a and b. The scan rates were inspected at 20, 40, 60, 80 and 100 mV.s^-1^. The results showed that cathodic peaks at 0.543 V and 0.645 V for both composites-based sensors are related to lactone ring reduction of C_6_-HSL for quasi-reversible systems. As the scan rate increased, the reduction peak current of C_6_-HSL increased without shifting towards more negative values. Additionally, there was a concurrent increase in the peak current, indicating a parallel escalation^66^. The correlation coefficients (R^2^) of 0.96 and 0.952 confirm a linear association between cathodic peak currents (i_pc_) and the square root of scan rate (ν ½) for both composites-based sensors, ZnO/PANI-DSBA and Fe_2_O_3_/PANI-DSBA as seen in Figs. 5c and d**,** according to the following equations^70^:

2 $$ {\text{For ZnO}}/{\text{PANI}} - {\text{DSBA based sensor}};{\text{ i}}_{{{\text{pc}}}} \left( {{\text{mA}}} \right) \, = \, - 0.0{\text{71 V}}\left( {{\text{mV}}.{\text{s}}^{{ - {1}}} } \right) \, + \, 0.0{189} $$

3 $$ {\text{For Fe}}_{{2}} {\text{O}}_{{3}} /{\text{PANI}} - {\text{DSBA based sensor}};{\text{ i}}_{{{\text{pc}}}} \left( {{\text{mA}}} \right) \, = \, - 0.0{\text{34 V}}\left( {{\text{mV}}.{\text{s}}^{{ - {1}}} } \right) \, + \, 0.0{141} $$

The obtained slopes of 0.487 and 0.495, were derived from plotting log i_pc_ (mA) against log scan rate (mV.s^-1^) as seen in Figs. 5e and f for both composites-based sensors. This proximity to 0.5 as described by the following equations^71^:

4 $$ {\text{For ZnO}}/{\text{PANI}} - {\text{DSBA based sensor}};{\text{ log i}}_{{{\text{pc}}}} \left( {{\text{mA}}} \right) \, = \, 0.{\text{487 log V }}\left( {{\text{mV}}.{\text{s}}^{{ - {1}}} } \right) \, - { 1}.{1} $$

5 $$ {\text{For Fe}}_{{2}} {\text{O}}_{{3}} /{\text{PANI}} - {\text{DSBA based sensor}};{\text{ log i}}_{{{\text{pc}}}} \left( {{\text{mA}}} \right) \, = \, 0.{\text{495 log V }}\left( {{\text{mV}}.{\text{s}}^{{ - {1}}} } \right) \, - { 1}.{ 48}. $$

These results suggest a diffusion-controlled sensor quasi-reversible system. So, this implies an improvement in ions transport to the inner electrolyte of both composite-based films; ZnO/PANI-DSBA and Fe_2_O_3_/PANI-DSBA^66,70,72^.

**Figure 6:**
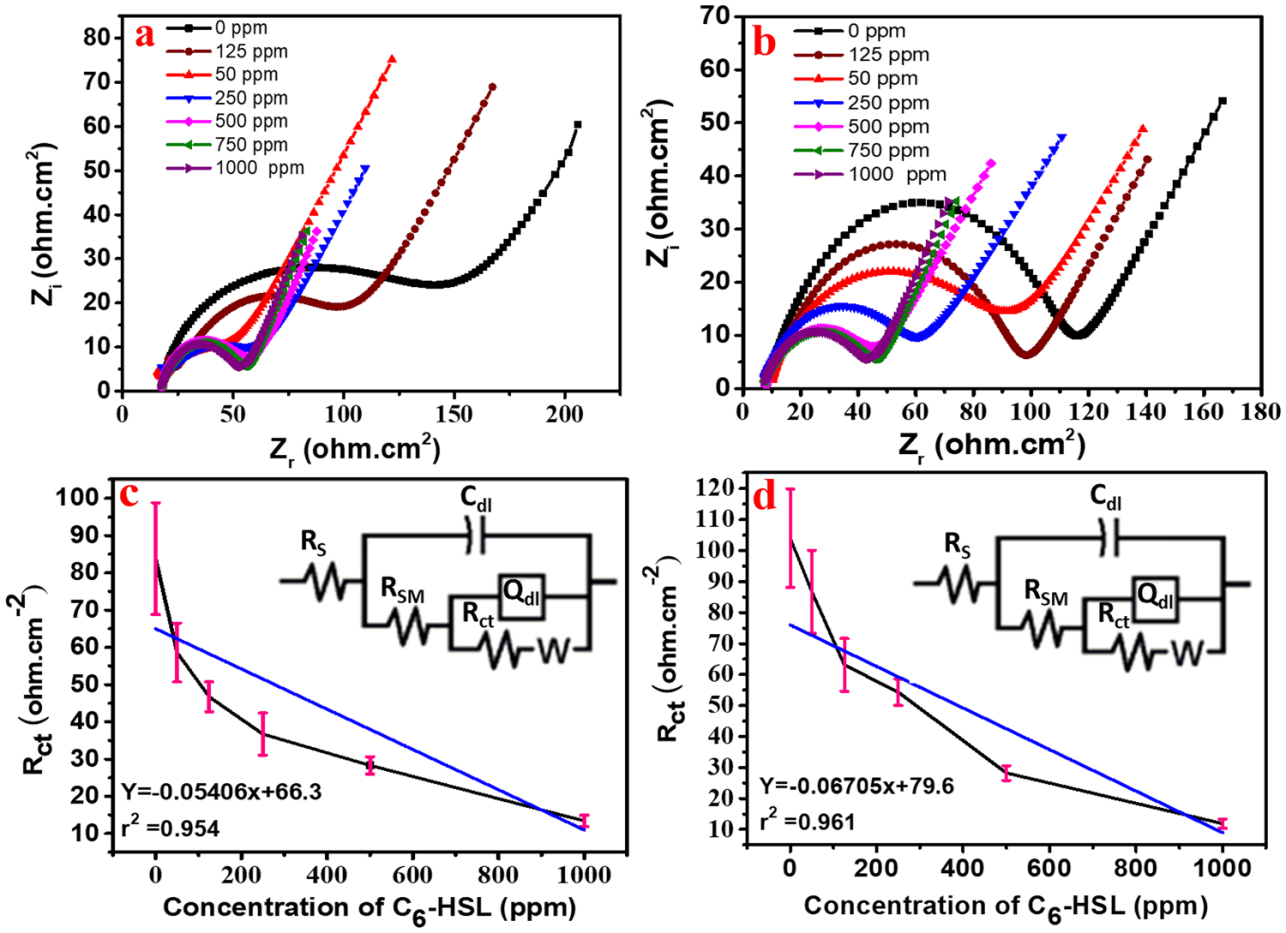
EIS Nyquist diagrams of (**a**) ZnO/PANI-DSBA based sensor, (**b**) Fe_2_O_3_ /PANI-DSBA based sensor immersed in the saline solution (2.63% NaCl) with the different doses of C_6_-HSL. (**c**) and (**d**) the Calibration curve of C_6_-HSL obtained with a frequency range of 0.01Hz-100 kHz and an amplitude of 150 mV for sensors based on ZnO/PANI-DSBA and Fe_2_O_3_/PANI-DSBA respectively.

To calibrate the C_6_-HSL amount per part per million (ppm), the known concentrations were dissolved in the prepared salty water then the electrochemical impedance spectroscopy (EIS) technique was implemented to measure the resistance for every C_6_-HSL dose. A water's salinity content was adjusted at 26,000 ppm (NaCl concentration) to simulate the salinity of the formation water sample where the total dissolved salts (TDS) of SRB incubated true sample was 2.6%. Additionally, other parameters like temperature or pressure are typically the same. The solutions for the calibration curves C_6_-HSL were prepared in the first step by dissolving it in a polar solvent that is miscible with water such as ethanol, and then the obtained solution was poured into 50 ml of salty water. The concentrations of the C_6_-HSL that were used for extrapolating the calibration curve varied from 50 to 1000 ppm. The total true resistance (Z_R_) was a sign of the C_6_-HSL concentration changing. The ZnO/PANI-DSBA and the Fe_2_O_3_/PANI-DSBA sensors were used for the calibration. The EIS spectra of the known concentrations of the C_6_-HSL were provided from the calibration curve. The electrical equivalent circuit (EEC) was obtained from the fitted EIS (see Fig. 6a–d) The Electrochemical parameters of the EEC are defined as follows; R_SM_ is sensing materials resistance, R_ct_ signifies charge transfer resistance and *n* is CPE exponent and it has values ranging from − 1 to 1, and (*W)* is a Warburg impedance^73,74^.

The EEC parameters of the detected C_6_-HSL at different concentrations were obtained by the fabricated sensors based on both sensing materials, ZnO/PANI-DSBA and Fe_2_O_3_/PANI-DSBA (Table 3). The standard calibration curve was created by plotting the estimated R_ct_ (Y-axis) against known concentrations of the C_6_-HSL (X-axis). According to the enclosed Fig. 6c and d, the presence of the C_6_-HSL with various concentrations (50–1000 ppm) for both sensors resulted in a quasi-linear correlation with R_ct_ and the general equation was (Y = slope X + intercept), where the equations were (Y = -0.05406X + 66.3, r^2^ = 0.954) and (Y = Y = -0.06705x + 79.6, r^2^ = 0.961) for the ZnO/PANI-DSBA and the Fe_2_O_3_/PANI-DSBA-based sensors, respectively. The LOD for the C_6_-HSL was determined to be 739 and 484 ppm for the ZnO/PANI-DSBA and the Fe_2_O_3_/PANI-DSBA-based sensors, respectively. The used formula for LOD was equal to 3s/m where s represents the standard deviation of R_ct_ in the blank sample (n = 3), and m corresponds to the slope of the relative calibration curves of the C_6_-HSL. Table S1 exhibits a comparison between our work and recently reported nanomaterial-based electrochemical methods for the determination of quorum sensing molecules. Through our investigation, we discovered that our sensor exhibits greater accuracy and is more efficient in detecting the C_6_-HSL molecules. As shown from the calibration curves of both sensors, the R_ct_ values were decreased by increasing the C_6_-HSL concentrations^11,12^. The theory of determining the concentration of bacterial signals mainly relies on the formation of a complex between the bacterial signals and the different metals included in the composition of the two prepared composites, ZnO/PANI-DSBA and Fe_2_O_3_/PANI-DSBA. Briefly, it is well known that the C_6_-HSL contains a lactone group, and this lactone group contains an oxygen atom that has unpaired electrons. Simply, the oxygen atom donated these unshared electrons to the “d” or “f” orbitals of the metals embedded in the composites and formed a coordination bond as follows: 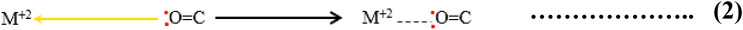

**Table 3:** Electrochemical parameters of EEC of the fitted Nyquist plots of the different sensing materials with different concentrations of C_6_-HSL in saline water (2.63% NaCl).

C_6_—HSL conc. (ppm)	Parameters							
	R_S_ (Ω.cm^2^)	C_dl_ (F.cm^−2^)	R_SM_ (Ω.cm^−2^)	Q (F.cm^−2^)	n	R_ct_ (Ω.cm^−2^)	W (Ω^-1^.cm^−2^ s^1/2^)	RSE (%)
ZnO/PANI-DSBA-based sensor								
0	1.97E + 01	3.20E − 06	1.44E + 02	3.84E − 04	4.07E − 01	8.38E + 01	6.05E − 03	11.31
50	2.17E + 01	6.93E − 04	1.35E + 02	1.92E − 04	5.76E − 01	5.86E + 01	8.20E − 03	13.50
125	1.09E + 01	5.32E − 03	9.43E + 01	4.48E − 04	5.41E − 01	4.67E + 01	7.14E − 03	8.55
250	7.87E + 00	1.72E − 08	6.71E + 01	3.86E − 04	5.10E − 01	3.67E + 01	1.09E − 02	10.54
500	7.68E + 00	2.20E − 06	4.22E + 01	4.22E − 04	5.10E − 01	2.83E + 01	1.94E − 02	8.29
750	7.13E + 00	3.94E − 6	4.01E + 01	2.93E − 04	5.01E − 01	19.3E + 01	1.56E − 02	10.15
1000	6.93E + 00	1.14E − 04	3.79E + 01	1.81E − 02	5.62E − 01	1.34E + 01	1.09E − 04	11.23
Fe_2_O_3_/PANI-DSBA-based sensor								
0	1.43E + 01	6.07E − 08	1.28E + 02	1.04E − 05	7.46E − 01	1.04E + 02	1.04E − 04	9.81
50	1.28E + 01	4.82E − 09	9.76E + 01	1.26E − 05	7.47E − 01	8.67E + 01	1.25E − 02	15.46
125	9.46E + 00	2.19E − 05	9.83E + 01	1.54E − 04	5.49E − 01	6.31E + 01	1.17E − 02	13.48
250	6.24E + 00	1.07E − 06	5.29E + 01	3.10E − 04	4.98E − 01	5.43E + 01	1.18E − 02	7.88
500	7.95E + 00	2.77E − 06	5.29E + 01	1.88E − 04	6.56E − 01	2.82E + 01	1.33E − 02	8.47
750	1.14E + 01	1.93E − 05	4.21E + 01	1.56E − 04	5.23E − 01	1.69E + 01	1.76E + 02	11.42
1000	6.45E + 00	1.15E − 04	3.79E + 01	1.72E − 02	5.77E − 01	1.19E + 01	1.17E − 03	12.77

The formation of a coordination complex between the lactone group as a dentate, and the metals as ligands, led to an enhancement of the interaction at the working electrode/metals interface. That means the greater the bacterial growth, the greater the formation of the complex via the sensor/medium interface. Therefore, the R_ct_ was diminished with the C_6_-HSL increment.

##### Selectivity of the fabricated sensors

**Figure 7:**
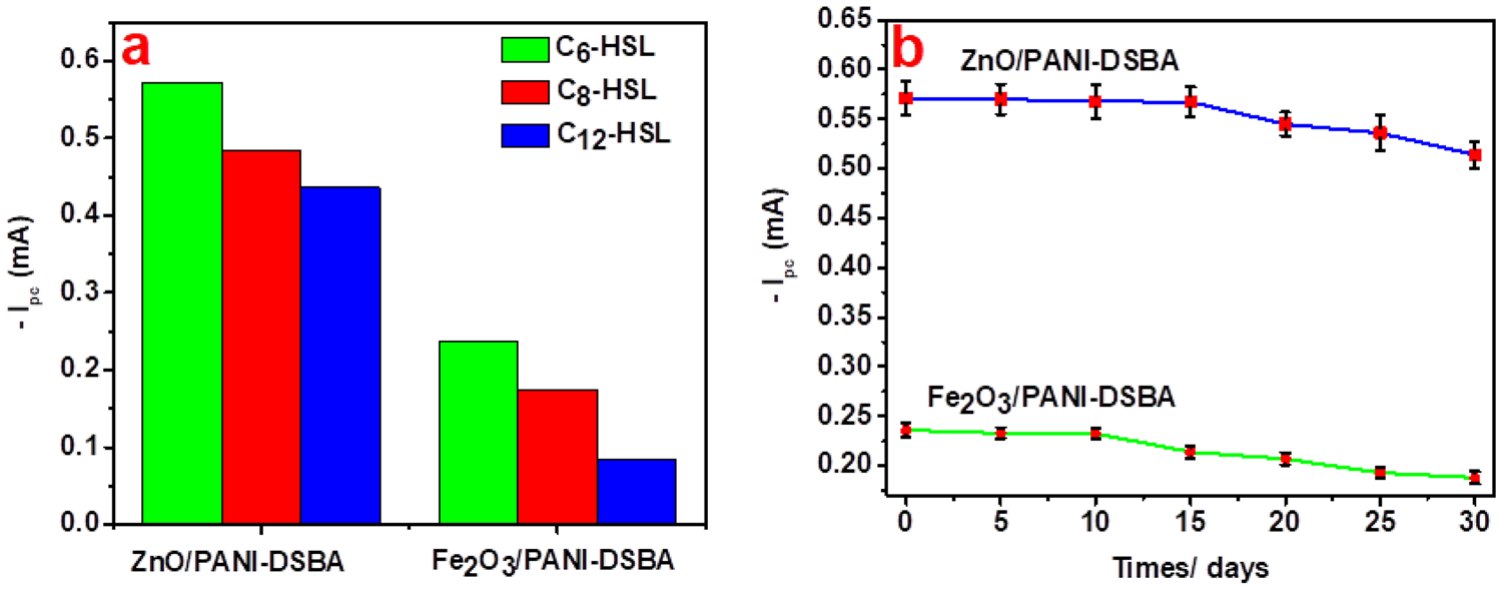
(**a**) Fabricated sensors selectivity via various AHLs; C_6_-HSL, C_8_-HSL, and C_12_-HSL at concentration 1000 ppm. (**b**) Stability test of ZnO/PANI-DSBA/CC and Fe_2_O_3_/PANI-DSBA/CC stored under N_2_ atmosphere at 25 °C (*n* = 3).

For assessing sensor selectivity, various AHLs, including C_6_-HSL, N-octanoyl-L- and N-Lauroyl-L- homoserine lactones (C_8_-HSL and C_12_-HSL), were examined by measuring i_pc_ values as a parameter indicating the sensitivity of the prepared composites. Figure 7a displays the i_pc_ values for various AHLs at a concentration of 1000 ppm, determined via CV using ZnO/PANI-DSBA and Fe_2_O_3_/PANI-DSBA-based sensors with a scanning rate of 50 V.s^-1^. Observations from the Figure indicate that, overall, the sensitivity of the ZnO/PANI-DSBA-based detector is higher than that of the Fe_2_O_3_/PANI-DSBA-based detector towards AHLs. This is evidenced by the higher i_pc_ values of ZnO/PANI-DSBA compared to Fe_2_O_3_/PANI-DSBA for each investigated AHL. This difference can be attributed to the greater ability of ZnO/PANI-DSBA to reduce the lactone ring of AHLs compared to Fe_2_O_3_/PANI-DSBA. Moreover, both sensors demonstrated increased sensitivity towards C_6_-HSL compared to other AHL molecules, with the maximum i_pc_ of 0.571 mA observed with the ZnO/PANI-DSBA-based sensor. This variance in i_pc_ is associated with the AHL molecule's ability to diffuse through the sensitive materials, facilitating ion transfer across the electrolyte/WE interface. Consequently, smaller-sized molecules exhibit enhanced penetration and increased conductivity, leading to maximized peak currents. Therefore, both fabricated sensors are more sensitive and selective in detecting C_6_-HSL molecules.

##### Sensors’ stability

The precision and practicality of the proposed method were validated by examining the reproducibility and storage stability of the ZnO/PANI-DSBA and Fe_2_O_3_/PANI-DSBA using CV. The findings, which affirm the reliability of the method, are illustrated in Fig. 7b. The fabricated sensors exhibited a relative standard deviation (RSD) of 3.4% for ten consecutive measurements of the response to 1000 ppm C_6_-HSL. For a span of 20 days, there was no discernible decrease in i_pc_ observed for the ZnO/PANI-DSBA-based sensor, while the Fe_2_O_3_/PANI-DSBA-based sensor exhibited lower stability, with i_pc_ values diminishing after 15 days. Over time, ZnO/PANI-DSBA proved to be more stable than Fe_2_O_3_/PANI-DSBA, attributed to the higher propensity of Fe^3+^ to reduce to Fe compared to the reduction of Zn^2+^ to Zn in the continuous cycle of oxidation from PANI-emeraldine to salt to PANI-pernigraniline salt alongside the reduction of metal oxide to metal. This observation aligns with the electrochemical series, confirming the greater activity of Fe^n+^ ions in reducing Fe metal compared to reducing Zn^n+^ to Zn metal. After 1 month, both sensors retained approximately 85–90% of their initial current, indicating remarkable reproducibility and stability. This can be attributed to the excellent compatibility and stability of their constituent layers: the CC layer, metal oxides layer, and PANI-DSBA layer. Moreover, the rapid charge transfer ability contributes to the response mechanism of the fabricated sensors to C_6_-HSL molecules.

##### Real sample measurements

**Figure 8:**
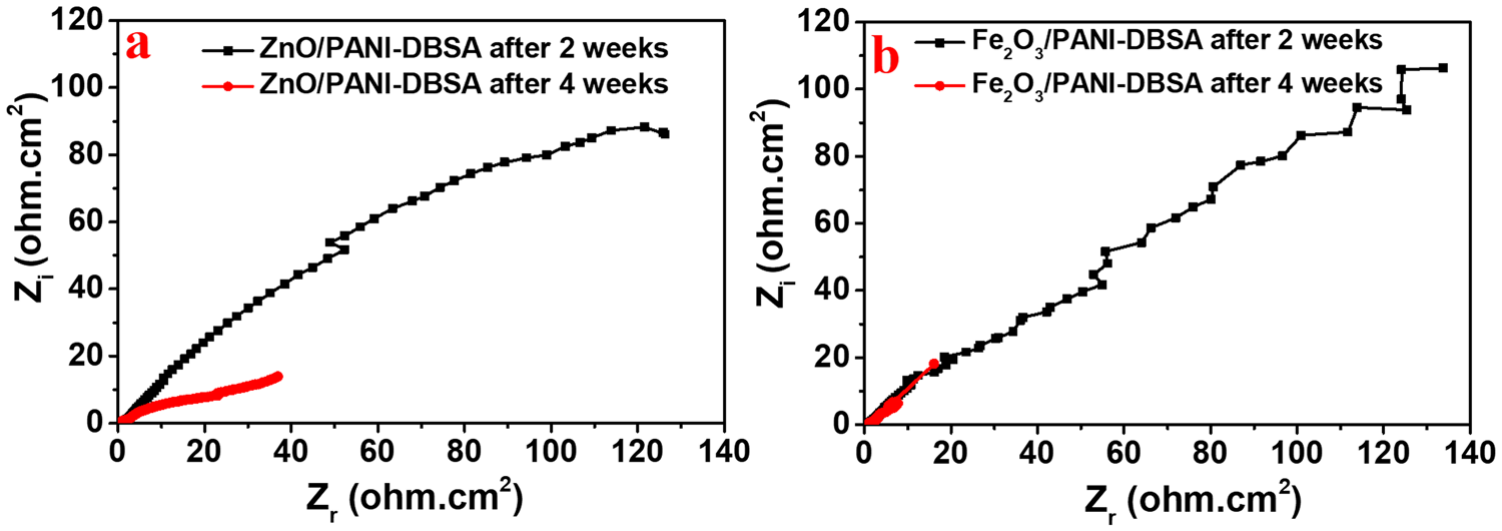
Performance of the prepared electrochemical sensors based on (**a**) ZnO and (**b**) Fe_2_O_3_ /PANI-DBSA composites to measure the C_6_-HSL concentrations in the formation water sample containing SRB over time (2 to 4 weeks).

To determine the different concentrations of the C_6_-HSL after SRB inoculation for varied periods (2—4 weeks). The SRB bacteria were allowed to be enriched and then the produced C_6_-HSL signals were estimated by the fabricated sensors. The measured unknown concentrations (ppm) of the C_6_-HSL presented in the formation water of oil wells, containing SRB bacteria with the RSD, can be seen in Table 4**,** where every sample was tested three times. As seen in Fig. 8a and b for both ZnO/PANI-DSBA and Fe_2_O_3_/PANI-DSBA based fabricated sensors, the bacteria count increased by time and relatively the C_6_-HSL signals as well increased, which means an increase in the conductivity as well as diminishing the resistivity. These findings confirmed the occurrence of the naturally developed biofilm, in the collected formation water, and displayed its impact on carbon steel, as observed in the previous section. It was noted that the C_R_ increased over time due to bacterial growth and biofilm formation. The results also confirmed the increment of the C_6_-HSL over time (from 2 to 4 weeks).

**Table 4:** The various concentration of C_6_-HSL (ppm) that were measured by various sensors at different measuring times.

Time of sensing by different sensors	R_ct_ value (Ω.cm^−2^)	C_6_-HSL concentration (ppm)	n	RSD
ZnO/PANI-DSBA after 2 weeks	3.42E + 01	570.2	3	± 2.97
ZnO/PANI-DSBA after 4 weeks	0.94E + 01	< 1000	3	± 1.54
Fe_2_O_3_/PANI-DSBA after 2 weeks	5.36E + 01	333.7	3	± 6.71
Fe_2_O_3_/PANI-DSBA after 4 weeks	0.53E + 01	< 1000	3	± 1.86

A similar attitude of both sensors was attributed to the presence of metal oxide on the fabricated sensors where they follow the capacitance behavior. On the other hand, when we measured the unknown concentrations of the actual sample after 2 and 4 weeks of growth time with the sensor fabricated only with PANI-DBSA, the performance of the sensor still followed the role of conductivity of SRB-solution that increased with the bacteria incubation period (see Fig. S10). This is explained by the ability of the PANI-DBSA to interact with a carbonyl group of the C_6_-HSL and form amide bonds. However, the pattern of Nyquist-EIS significantly differed from the sensors containing composites of the PANI-DSBA or the Fe_2_O_3_ or ZnO. In conclusion, both sensors observed the same attitude to detect the C_6_-HSL in the contaminated formation water with a slight superiority to the ZnO/PANI-DBSA, this may be attributed to the electrocatalytic activity and fast electron transfer properties of the ZnO as well as its nanomorphology.

### Colorimetric confirmation of C_6_-HSL

**Figure 9:**
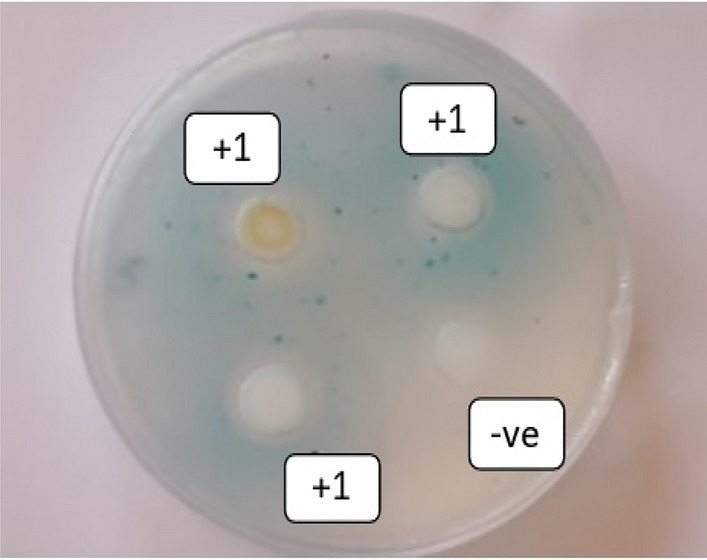
Colorimetric sensing of C_6_-HSL of SRB bacteria under ambient conditions. + ve (positive control), − ve (Negative control), + 1 (medium intensity of the C_6_-HSL producing SRB).

The C_6_-HSL production from the enriched-SRB biofilm was calorimetrically calculated and displayed in Fig. 9. The result was calculated as the C_6_-HSL intensity i.e. + 1 which indicates the medium quantity of the C_6_-HSL production^75^. It has been reported that SRB-biofilms use the C_6_-HSL signal molecules for metabolic activity and interspecies communication^76^. Furthermore, Sivakumar et al. (2019) described a potential correlation between the C_6_-HSL intensity and SRB-biofilm in a salinity medium^14^. Moreover, the increased expression of biofilm-related genes and SRB-enzyme`s productivity were linkages between the produced C_6_-HSL and transcriptomic sulfate^14^.

## Conclusions

In conclusion, the synthesis and characterization of two MO_x_/CPs nanocomposites, namely ZnO/PANI-DBSA and Fe_2_O_3_/PANI-DBSA, were accomplished using various analytical techniques. These nanocomposites were utilized in the detection of C_6_-HSL through electrochemical analysis, where the ZnO/PANI-DBSA-based sensor exhibited higher sensitivity compared to Fe_2_O_3_/PANI-DBSA. The detection limits for C_6_-HSL were found to be 624 ppm and 441 ppm for ZnO/PANI-DBSA and Fe_2_O_3_/PANI-DBSA, respectively, with a linear range of 50–1000 ppm. Furthermore, electrochemical assessment using SPE after SRB incubation periods revealed a decrease in R_ct_ values with increasing C_6_-HSL concentrations, indicating enhanced conductivity due to SRB-biofilm formation. The colorimetric assessment confirmed the correlation between SRB-biofilm growth under salinity conditions and C_6_-HSL intensity. The utilization of ZnO/PANI-DBSA and Fe_2_O_3_/PANI-DBSA in the SPE provides a practical and advantageous method for accurately measuring C_6_-HSL concentration in oil & gas wells, offering a simpler and more cost-effective alternative to existing methods. Moreover, the CV analysis demonstrated that both sensors operate through a diffusion-controlled quasi-reversible system. Selectivity testing revealed higher sensitivity of ZnO/PANI-DSBA-based detector towards AHLs compared to Fe_2_O_3_/PANI-DSBA-based detector, with increased sensitivity towards C_6_-HSL. Moreover, both sensors showed heightened sensitivity to C_6_-HSL compared to other AHL molecules, with the ZnO/PANI-DSBA-based sensor achieving a maximum i_pc_ of 0.571 mA. Stability testing indicated superior stability of ZnO/PANI-DSBA over Fe_2_O_3_/PANI-DSBA, with both sensors maintaining approximately 85–90% of their initial current after one month, highlighting remarkable reproducibility and stability.

### Supplementary Information

Supplementary Information.

## Data Availability

The datasets used and/or analyzed during the current study are available from the corresponding author upon reasonable request.
