# Supplementary material for: Detectable quorum signaling molecule via PANI-metal oxides nanocomposites sensors
Source: Sci Rep. 2024 May 2;14:10041. doi: 10.1038/s41598-024-60093-8 (PMC11063039; doi:10.1038/s41598-024-60093-8)
Supplement: Supplementary file 1 — Supplementary Information. [file 41598_2024_60093_MOESM1_ESM.docx]

**Detectable Quorum Signaling Molecule via PANI-Metal Oxides Nanocomposites Sensors**

**Walaa S. Gado^1*^, AbdAlrahman G. Al-Gamal^1*^, Mona Shaban E. M. Badawy^2^, A. Labena^1^, Khaled Zakaria^1^,** **Khalid I. Kabel^1^**

^1^Egyptian Petroleum Research Institute (EPRI), Nasr City 11727, Cairo, Egypt.

^2^ Department of Microbiology and Immunology, Faculty of Pharmacy (Girls), Al-Azhar University, Cairo, Egypt.

*Corresponding authors: E-mails: [abdoepri@yahoo.com](mailto:abdo2030@yahoo.com), [Walaa_shabaan86@yahoo.com](mailto:Walaa_shabaan86@yahoo.com).

^*^These authors equally contributed to this work.

c

1. **Experimental work**
   1. *Raw materials and characterization apparatus*

N-Hexanoyl-L-homoserine lactone [C_6_-HSL, 99%], Zinc acetate dehydrate [Zn(CH₃CO₂)₂. 2H_2_O, 98%], ferric chloride hexahydrate [FeCl_3_.6H_2_O, 99% ], Sodium chloride [NaCl, 99,9%], and Aniline [C_6_H_5_NH_2_, 99.5%] were purchased from Sigma Aldrich. Dodecylbenzene sulfonic acid [BSA, 70 wt. %], Potassium persulfate [KPS, 99.9%], Polyethylene glycol [PEG-4000, PEG-400] and Polyvinyl pyrrolidone [PVP] were supplied from Merck, Ammonia solution [NH_4_OH, 28-30%], Methanol, Butanol, Hexane, and Acetone were purchased from Honeywell Burdick & Jackson.

The synthesized metal oxides nanoparticles (MO_x_ NPs) and their polyaniline-dodecyl benzene sulphonic acid (PANI-DBSA) nanocomposites were characterized via X-ray Powder Diffraction (XRD) (X’Pert Pro, Malvern Co., UK) 2θ: 5ᵒ - 90ᵒ, λ = 1.5406 Å. The morphology of the synthesized materials was characterized using High-Resolution Transmission Electron Microscopy (HRTEM) (JEOL JEM-2100F, Japan, 200 KV) and Field-emission Scanning Electron Microscopy (FE-SEM; JEOL, JSM-6700F, Japan, acceleration voltage; 500 V to 300 KV). Zeta Potential of the prepared materials (ZnO and Fe_2_O_3_ NPs) was obtained from Dynamic Light Scattering (DLS, Zetasizer Nano-ZS90 instrument, Malvern Co., UK). The distinct functional groups of the prepared PANI-DBSA were characterized using FT-IR Spectroscopy (Thermo Fisher Scientific, USA). The disordered in the PANI-DBSA and its composites were observed by dispersive Raman spectroscopy (Senterra, Germany) where the laser source was, He-Ne 3000 with laser wavelength 632.8 nm, equipped with a CCD detector, and the samples were maintained at room temperature throughout the experiments. The obtained emulsified polymer molecular weight (M_wt_) was detected by Gel Permeation Chromatography (GPC) (Agilent, THF model 600E, USA, GPC-water 2410, reflective index detector styragel HR THF 7.8 × 300 mm, PS standard). Computer numerical control machine (CNC) SIM-KIT 40*60 CS, Simplex, Egypt was used for sensor fabrication and chip design. The electrical measurements were monitored using computer-controlled Origa Flex-Pack OGF01A/potentiostat-galvanostat (Origalys, France). The system was planned by Origamaster 5 software V2-4-0-4.

- 1. *Synthesis of MOx NPs*

ZnO was prepared as described elsewhere [1]. Briefly, add drops of alkali solution of NH4OH to a salt solution of Zn (CH₃CO₂) ₂. 2H_2_O until reaching a basic pH value, after which the precipitate was washed and filtered several times and then dried for a while until a precipitate was obtained and roasted at certain temperatures.

Fe_2_O_3_ was synthesized at room temperature as follows; a transparent NaOH solution was synthesized by dissolving 32 g of sodium hydroxide in 1 litre of distilled water. Another solution was made by adding 42 g of FeCl_3_.6H_2_O to 200 ml of distilled water and kept under stirring for 15 min. Following that, drop by drop of FeCl_3_.6H_2_O solution was added to a NaOH solution under stirring conditions. The pH was kept slightly alkaline at 8 to enhance the brownish precipitate of ferric hydroxide. 1 h later, the precipitate was filtered and dried for 18 h at 80°C. The precipitate was annealed at 450°C for 10 h, slowly cooled down to room temperature, and eventually rinsed with deionized water until the pH approached 7 to precipitate of Fe_2_O_3_ brownish [2].

- 1. *Sensor fabrication*

The electrochemical sensor is based on an advanced integrated structural using a screen printed technique supported by a three-electrode design, the printed circuit board (PCB) was designed via CNC machine through digital software for a signal amplification function. The interdigitated electrodes were fabricated using silver ink (Ag/AgCl) as a reference electrode (RE), the carbon paste over Cu plate was used as a counter electrode (CE), and the sensing layer, deposited of PANI-DBSA nanocomposites, as a working electrode (WE). The WE was prepared by mixing the conductive carbon paste with the prepared sensitive materials ≤ 1% (w/w) then spread uniformly over the Cu electrode via drop casting. Afterwards, they were then permitted to dry under a vacuum while remaining at ambient temperature for 24 h. Finally, an insulating layer was deposited, see **Figures** **S1a and b**. The sensor design was usefully achieved using the Autodesk software, with dimensions of 1 cm^2^ from all directions, while the Protus software was applied for the electrical circuit design. The sensor was fabricated via a laser engraving technology with specific dimensions, a 4-axis control with built-in motion control CPU through the Mach3 software, on the surface of a PCB to form a three-electrode electrochemical chip [3, 4].

- 1. *Colourimetric detection of C_6_-HSL*

The biosensor Agrobacterium tumefaciens KYC55 was growing on AT minimal salt medium. The medium was first prepared as 20×buffer (KH2PO4 214 g/l at pH 7.0), 20× salts ((NH_4_)_2_SO_4_ 40 g/l, MgSO_4_-7H_2_O 3.2 g/l, CaCl_2_- 2H_2_O 0.2 g/l, MnSO_4_-H_2_O 0.024 g/l), 100× Iron stock (22mM of FeSO_4_-7H_2_O 0.008 g/100ml and Glucose 50 g/ 100 ml. A 100 μg/ ml of Gentamicin, 100 μg/ml of Spectinomycin, and 4 μg/ml of Tetracycline were supplemented to the AT minimal medium to maintain the A. tumefaciens strain with the convenient plasmids [4]. Agar was added to the AT minimal medium as a solidifying agent. The C_6_-HSL signal molecules were extracted from the enriched-SRB and were propagated in the saline medium by the method mentioned by [5]. C_6_-HSL was examined using the agar well-diffusion assay method [6]. The biosensor strain Agrobacterium tumefaciens was streaked over the agar plates then the agar surface was cut into 6 mm wells using a sterile borer. Afterwards, the enriched SRB C_6_-HSL extract, 60 μl, was added into wells. After that, the plates were incubated overnight at 28°C. Hexanoyl-L-homoserine lactone (Sigma-Aldrich, Germany) at a concentration of 50 μg/ml was used as a positive control. Ethyl acetate was used as a negative control. The appearance of the blue colour indicated C_6_-HSL generation in the provided samples.

1. **Result and discussion**
2. *Characterizations of the prepared PANI-DBSA, MO_x_ and composites*

The FTIR spectrum **Figure S3** of PANI-DBSA provides information about the molecular structure and functional groups present in the prepared composite. The peaks at the wavenumbers 3430 cm^-1^ and 3224 cm^-1^ reflect the presence of N-H stretching vibration, specifically in the amine and imine groups of PANI-DBSA. The peaks at 1550 cm^-1^ and 1490 cm^-1^ correspond to the stretching vibration of the C=N and C=C. Especially 1490 cm^-1^ indicates the presence of conjugated double bonds, which is a characteristic feature of conductive polymers. The peak at 1342 cm^-1^ indicates the presence of C–N stretching of the secondary amine of PANI backbone in the composite matrix which is likely attributed to the aromatic rings of the polyaniline backbone. The peaks at 1180 and 580 cm^-1^ correspond to the stretching vibrations of the sulfonic groups (SO^3-^) present in the DBSA dopant. These peaks indicate the successful incorporation of the DBSA dopant into the PANI matrix. The absorption peak at 692 cm^-1^ is due to the aromatic C-H bending vibration, referring to the DBSA dopant in the polymeric matrix [7, 8]. The SEM image (**Figure S4**) depicted the morphology of the powder sample of PANI-DBSA, revealing a polymer with a bulky structure and a uniform micro-porous arrangement.

The zeta potential and PDI values (see **Figure S5**) confirmed the homogeneity of the prepared MO_x_ with high crystallinity. Based on the information provided, it appears that the zeta potentials of the nanoparticles are within the range of +30 to -30 millivolts. This range indicates that the nanoparticles are stable and not aggregating in the medium they are dispersed in, which, in this case, is distilled water. The average size of the ZnO nanoparticles is 39 nm and 43 nm for Fe_2_O_3_. These size measurements indicate the average dimensions of the nanoparticles in each respective type. The stability of the particles was proved to be good as the zeta potential existed in the -23 mv range.

The Raman spectrum analysis reveals important information about the molecular structure and interactions within the samples studied. The Raman spectrum of the prepared PANI-DBSA was presented in (**Figure S6a**). The characteristic bands of the PANI-DBSA include peaks at 1110 cm^−1^ (C-H bending), 1200 cm^−1^ (C-N stretching), 1480 cm^−1^ (C=N stretching), 1560 cm^−1^ (N-H bending), and 1598 cm^−1^ (C=C stretching). In the case of PANI-DBSA spectra, characteristic bands were observed at 1198 cm^−1^ (C-H bending), 1210 cm^−1^ (C-N stretching), 1392 cm^−1^ (C-N^+^ stretching), 1480 cm^−1^ (C-C stretching). The conductive nature of PANI-DBSA can be attributed to the presence of a band located at 980 cm^−1^, which corresponds to the deformation of C-N in the secondary amine. In **Figure S6b**, the Raman spectrum of pure ZnO displayed three primary peaks. One prominent peak was observed at approximately 440.5 cm^−1^, which indicated a characteristic vibration of ZnO. Another weaker peak was present at 550 cm^−1^, while a third, even weaker peak was observed at 426 cm^−1^. These peaks corresponded to specific vibrational modes of ZnO. In **Figure S6c**, the Raman spectrum represented the ZnO/PANI-DBSA composite. Additional new peaks were detected at 1590 cm^−1^, 1360 cm^−1^, and 1150 cm^−1^. These peaks originated from the C–C stretching vibrations associated with the conjugation of PANI. The interaction between ZnO and the PANI-DBSA matrix amplified these peaks. Comparing the composite spectrum with that of pure ZnO, it was evident that the intensities of the peaks at 440 cm^−1^ in the composite were significantly reduced. Additionally, the peak at 426 cm^−1^ shifted to around 350 cm^−1^, and the peak at 566 cm^−1^ almost disappeared. These changes suggested that the density of defects within the composite was lower than that in pure ZnO. This improvement in defect density was attributed to the interaction between ZnO and the PANI-DBSA matrix. Furthermore, the presence of a peak at 1150 cm^−1^ indicated the existence of C–H bonding in the hybrid structure, suggesting possible atomic/molecular linkages between ZnO and PANI. The identification of PANI peaks and the observed shifts in the characteristic peaks of ZnO in the ZnO/PANI-DBSA composite confirmed the presence of an interaction between ZnO and the PANI matrix at a molecular level. **Figure S6d** has presented the Raman spectrum of Fe_2_O_3_ NPs, it exhibits three strong peaks located at approximately 224 cm^−1^, 285 cm^−1^, and 410 cm^−1^ as well as a weak peak at 605 cm^−1^. Both peaks at 285 cm^−1^ and 410 cm^−1^ are associated with the stretching (Fe–O) mode between two Fe and O atoms. However, after doping with PANI-DBSA (**Figure S6e**), the intensity of these peaks decreases, and new peaks appear at 1492 cm^−1^ (C=N stretching of the quinoid rings), and 1589 cm^−1^ (C-C stretching of benzenoid rings). Additionally, two distinct peaks at 1492 cm^−1^ and 1589 cm^−1^ can be attributed to the stretching vibrations of C-N^+^ fragments, indicating the presence of PANI. These results indicate that PANI is doped on the Fe_2_O_3_ NPs.

1. *Electrochemical Assessment of enriched-SRB biofilm using SPE*
   - 1. *OCP data analysis*

It is well-known that OCP refers to the potential that has been formed between the working electrode and the medium in comparison with a reference electrode. **Figure S7** displayed the difference in the corrosion potential (*E_corr_*) of the carbon steel as a function of time in the sterile (control) and SRB-containing media, the data revealed that there is a shift of *E_corr_* in the direction of active value (negative direction) at –684 mV/SCE that happened in the first 5 days followed by a quick positive shift of *E_corr_* at values of–591.9 and –549.9 mV/SCE between two periods 5 and 15 days that can be due to the acceleration of the cathodic reaction affected by the presence of the enriched SRB [9]. Then, a slight shift in *E_corr_* toward a negative direction started at 20 days before a reasonably stable region until 30 days had occurred, indicating a metallic dissolution process. The change in the potential emphasizes that the performance and the growth of the SRB have increased and the redox interaction of the medium quickened the carbon steel deterioration in the so-called MIC [10]. Interestingly, this observation indicated that SRB has colonized, propagated, and adhered to the surface of carbon steel to form a biofilm [11]. From the MIC point of view, the aggressiveness factors of the biofilm and the active metabolisms of the enriched SRB change the electrochemical reaction operation. Consequently, multiple cathodic reactions insertion, pH level alterations, more sulfide output, and extracellular polymeric substances (EPS), involved in the matrix of biofilm, were all identified as contributing variables that progressed the electrochemical interaction at the metal surface/biofilm interface and at the biofilm environment itself [12].

Turning to the OCP values of the carbon steel soaked in a sterilized medium (abiotic controller system) at the same interval period, there was a prominent increase of the *E_corr_* occurred during the 15 days at– 613.3 mV/SCE followed by a partial decrease at 20 days being about– 626.6 mV/SCE which then remained steady at approximately –630.0 mV/SCE. The buildup of the growth medium ingredients including sodium chloride, potassium sulfate, and phosphorus on the metal surfaces may be one rationale for the probable change in the control sample [13]. Particular attention is paid to the difference in *E_corr_*, there was a rise in all OCP values for the carbon steel metal in both mediums during the testing period within the whole intervals until 15 days of immersion, but the observed shift in the medium containing enriched-SRB was the largest as compared to the one in the control medium (approximately of about, 65 mV/SCE). Above all, the positive shift in the *E_corr_* was known as an OCP ennoblement phenomenon which was considered the most notable process in the MIC investigations [14]. The OCP ennoblement phenomena may be caused by the accumulation of complex residues of microbial cells, EPS, organic compounds, and inorganic materials on the surface of the metal, which accelerated metal corrosion by altering the metal's electrochemical conduct [15].

- - 1. *EIS data analysis*

The EIS was performed at a stable corrosion potential (OCP) for a period of 30 days to analyze the corrosion behaviour, and a carbon steel electrode was immersed in both the controller sterilized medium and the inoculated medium with the enriched-SRB. The impedance spectra were curve-fitted with the ZSimpWin 3.10 version software satisfactorily (errors < 10%) for getting an evaluation of the electrochemical factors at the metal/electrolyte contact in terms of quantity. It can be noted from **Figures S8a and b** that all the obtained impedance spectra exhibited faintly distorted semi-circles, elucidating the electrochemical electrolyte/ metal exterior interface's non-ideal capacitance behaviour. It is commonly accepted that the distorted semi-circles in the Nyquist plots were attributed to the frequency dispersal, resulting from the roughness, heterogeneity, and mass transport process on the metal surface [16]. Therefore, pure double-layer capacitors were sufficiently expressed by a transfer function with CPE to reimburse for the non-ideal capacitive restraint at the interface getting a more exact fit of the investigational information set [17]. The CPE was related to (n) which is the indicator of the surface inhomogeneity and the following equation illustrates how the CPE's impedance function works [18]:

** …………………. (2)**

where, Yo is a relational factor; ω is the angular frequency; and, a variance parameter (–1 ≤ n ≤ +1), has a phase-shifting meaning. While n = 0, the CPE represents an ideal resistor, for n = –1 an inductor, and for n = +1, a pure capacitor. According to the following equations, the values of C_dl_ and the biofilm's capacitance (C_bf_) are calculated from the CPE values. [19]:

** ……………….. (3)**

** ………………… (4)**

As offered in **Figure S8**, it is evident that there is an observed remarkable difference in the diameter of the Nyquist loops for both sterile medium and SRB medium at all the interval periods of immersion time, elucidating that the corrosion progress was corroborated by the presence of the enriched-SRB [20]. In addition, it was noted that the diameter of the capacitive semicircle loop predominantly increased with the insertion time of the carbon steel in the case of the sterile medium, implying that the production of a surface layer due to steel oxidation in addition to the deposition of corrosion outcomes on an outer surface of the carbon steel [21]. This could be explained with the help of the well-known suggestion that these corrosion products become more compact on the surface of the substrate with the exposure time. To put it simply, a larger semicircle diameter in Nyquist plots commonly signifies more elevated electrical resistance at the metal/tested medium interface, emphasizing lower corrosion rates (C_R_) [22].


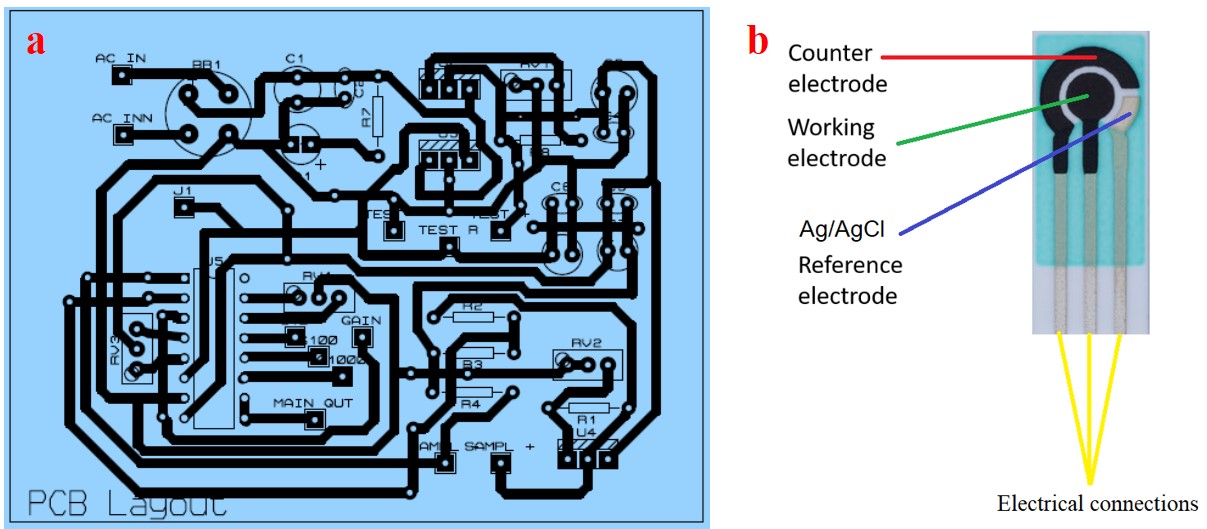


**Figure S1.** Lab-made PCB sensor for detection of the SRB-biofilm existence (a), circuit layout (b).

**
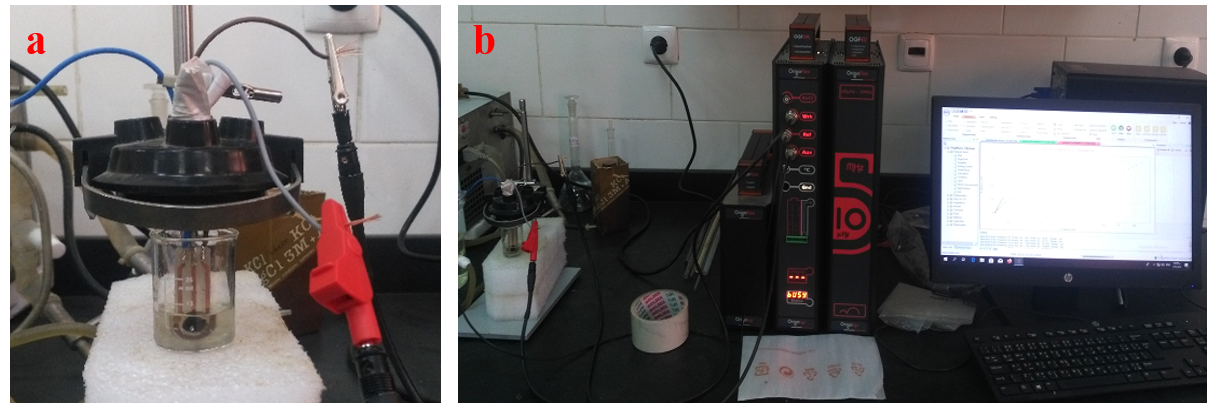
**

**Figure S2.** Electrochemical cell with fabricated sensor (a), the experimental setup for AHL detection (b).


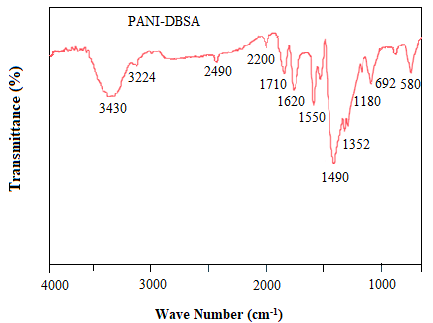


**Figure S3.** FT-IR spectrum of PANI-DBSA.

**
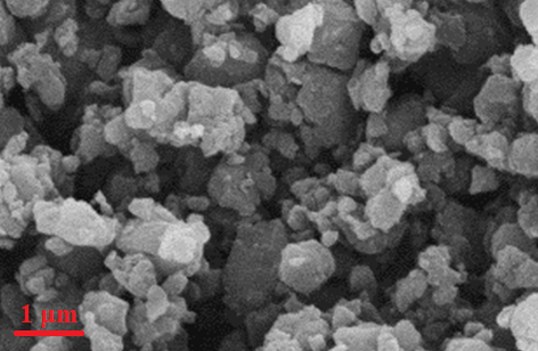
**

**Figure S4.** SEM image of PANI-DBSA overview.

**
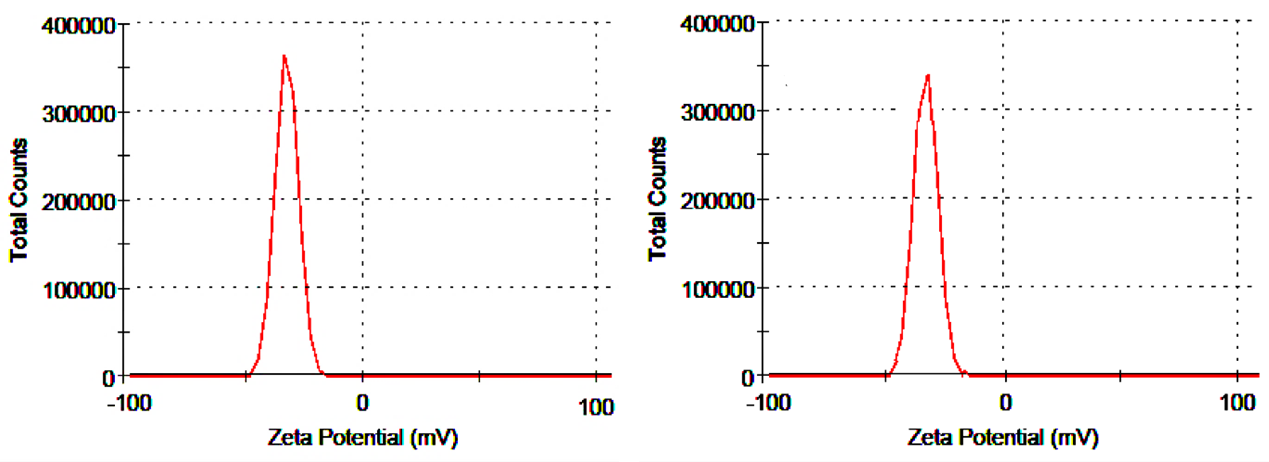
**

**Figure S5.** Zeta potential of ZnO NPs (a), Fe_2_O_3_ NPs (b).

**
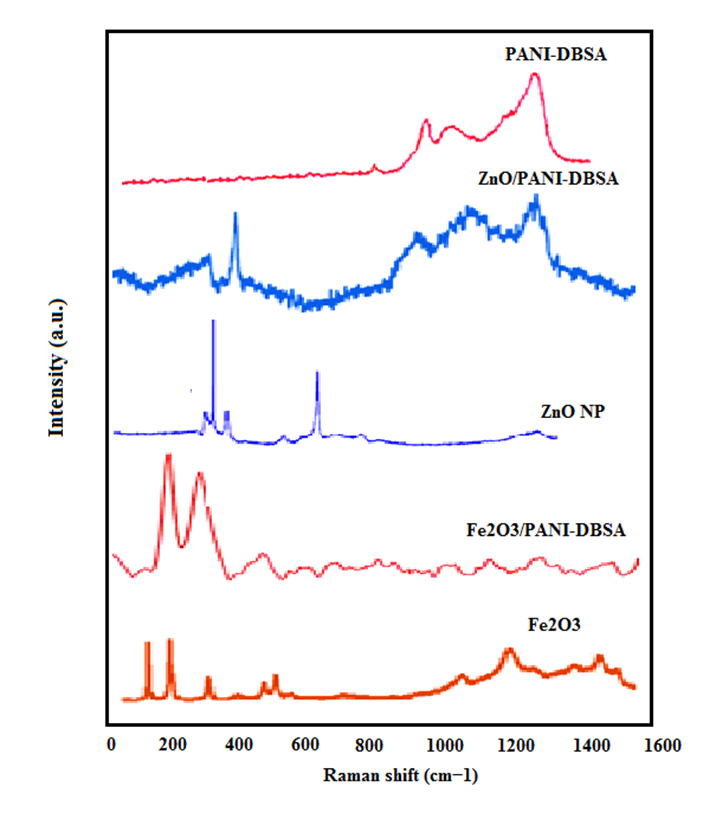
**

**Figure S6.** Raman spectrum of (a) PANI-DBSA, (b) ZnO NPs, (c) ZnO/ PANI-DBSA, (d) Fe_2_O_3_ NPs, and (e) Fe_2_O_3_/PANI-DBSA.


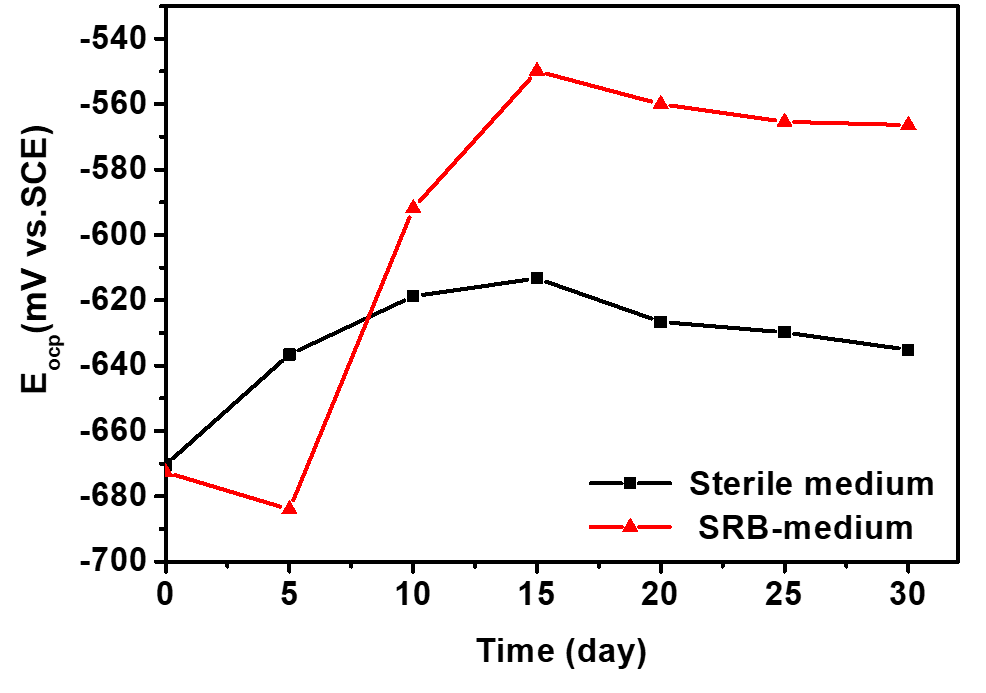


**Figure S7.** Time-dependence of OCP of carbon steel specimens in the tested media in the absence and presence of SRB


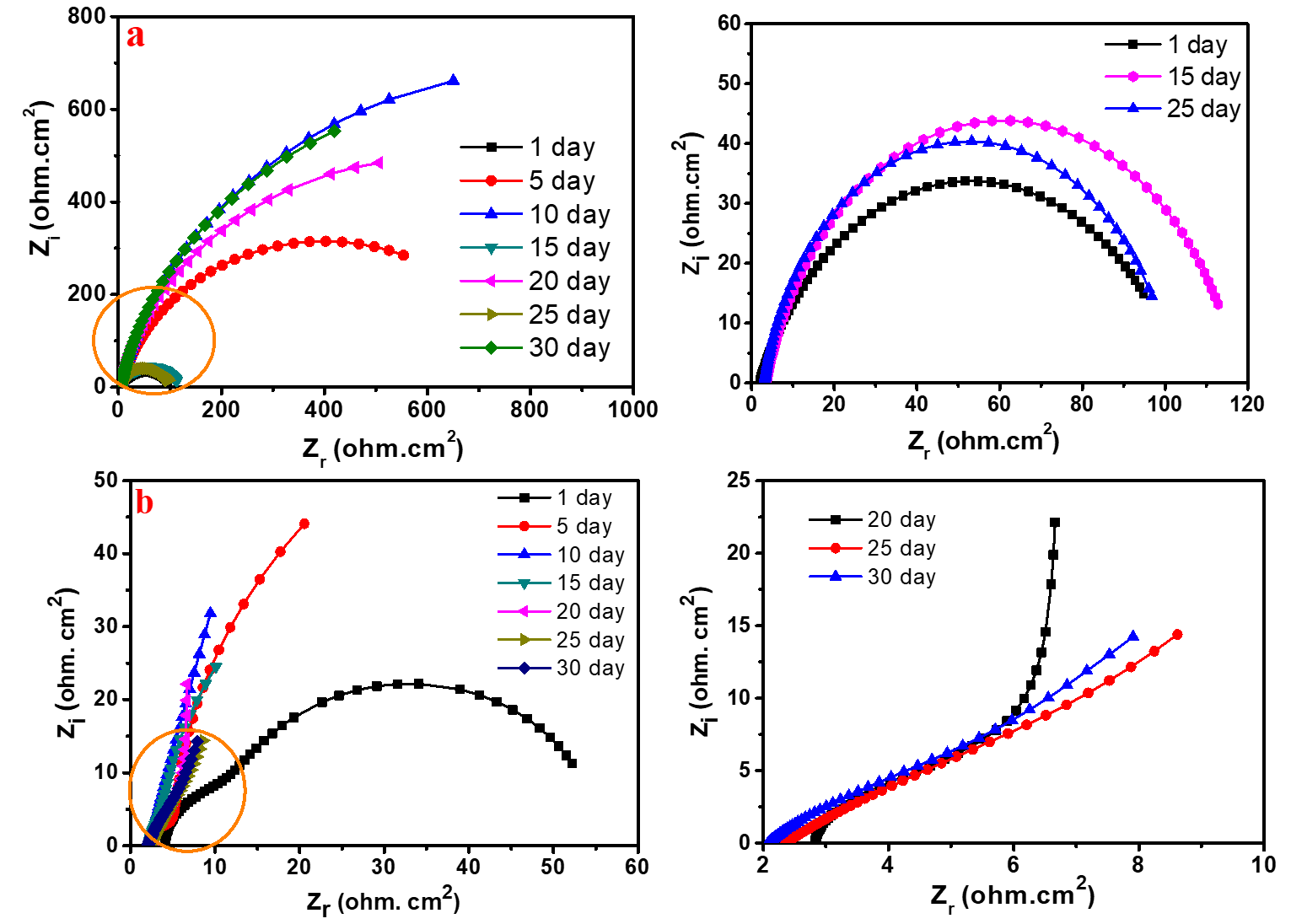


**Figure S8.** Nyquist plots of the carbon steel in bio-corrosive media during a period of 30 days of immersion; (a) sterile medium and (b) SRB-medium**.**

**Figure S9.** Equivalent circuit models used for fitting the experimental impedance data; (a) one-time constant (for sterile medium) and (b) Two-time constant (for SRB-medium).

**
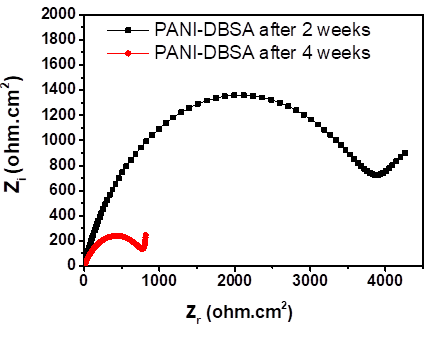
**

**Figure S10.** EIS of prepared electrochemical sensors based on PANI-DBSA with the change in bacterial growth time of SRB from 2 to 4 weeks.

**Table S1**: An overview of recently reported material-based electrochemical methods for the determination of quorum-sensing molecules

| Used Materials | Detection and Quantitative target | Detection Technique | LOD | Ref. |
| --- | --- | --- | --- | --- |
| RBL-2H3 mast cells- mast cells encapsulated in alginate/graphene oxide (NaAgl/GO) | N-3-oxododecanoyl homoserine lactone (3OC_12_-HSL) | EIS | 0.034 μM with a linear detection range of 0.1 to 1 μM | [23] |
| RBL-2H3 mast cells encapsulated in alginate/graphene oxide (NaAgl/GO) immobilized on the MWNTs/SPCE | N-3-oxododecanoyl homoserine lactone (3OC_12_-HSL) | EIS | 0.094 μM with a linear detection range of 0.1 to 1 μM | [24] |
| Fe_3_O_4_@SiO_2_- molecularly imprinted polymer (MIP) | C_4_-HSL, C_6_-HSL, C_8_-HSL, and N-3oxo-C_6_-HSL | DPV | 8×10^-10^ mol. L^-1^ with a linear detection range of 2. ×510^-9^ to 1.0 ×510^-7^ mol. L^-1^ | [25] |
| GCE/TEMPO–ZnPc | 3-oxo-C_12_-HSL | DPV | 1.8 × 10^–6^ mol dm^−3^ with linear detection range 2.32 × 10^–6^ and 39.9 × 10^–6^ mol dm^−3^ | [26] |
| ZnO/PANI-DBSA and Fe_2_O_3_/PANI-DBSA/carbon paste (CP) SPE sensor | C_6_-HSL | EIS | 624 and 441 ppm with a linear detection range of 50 to 1000 ppm | This work |

**References**

1. Labena A (2021) Novel, Low Cost and Fast Detection Sensor for Biogenic H2S Gas Based on Polyaniline/ZnO, CdO and CeO2 nanocomposites at Room Temperature*.* Egyptian Journal of Chemistry 64(6):3093-3104.

2. Lassoued A, Dkhil B, Gadri A, and Ammar S (2017) Control of the shape and size of iron oxide (α-Fe2O3) nanoparticles synthesized through the chemical precipitation method*.* Results in Physics 7:3007-3015. DOI: <https://doi.org/10.1016/j.rinp.2017.07.066>.

3. Kabel K, Al-Sabagh A, Sharara T, Badawi AM, Abdel-Rahman A, and Gado W (2020) Fabrication of H2S gas sensor based on PPy/CuO and PPy/SnO2 nanocomposites at room temperature*.* Egyptian Journal of Chemistry 63(7):2763-2774.

4. Ramadan Y, Amro H, Nora F, and Khaled AA (2017) Quorum sensing signal production by sponge-associated bacteria isolated from the Red Sea, Egypt*.* African Journal of Biotechnology 16(32):1688-1698.

5. Sivakumar K, Scarascia G, Zaouri N, Wang T, Kaksonen A, and Hong P (2019) Salinity-Mediated Increment in Sulfate Reduction, Biofilm Formation, and Quorum Sensing: A Potential Connection Between Quorum Sensing and Sulfate Reduction? Frontiers in Microbiology 10 DOI: 10.3389/fmicb.2019.00188.

6. Zhu J, Chai Y, Zhong Z, Li S, and Winans SC (2003) Agrobacterium bioassay strain for ultrasensitive detection of N-acylhomoserine lactone-type quorum-sensing molecules: detection of autoinducers in Mesorhizobium huakuii*.* Applied and Environmental Microbiology 69(11):6949-6953.

7. Kumar J, Shahabuddin M, Singh A, Singh S, Saini P, Dhawan S, and Gupta V (2014) Highly Sensitive Chemo-Resistive Ammonia Sensor Based on Dodecyl Benzene Sulfonic Acid Doped Polyaniline Thin Film*.* 6:1-8. DOI: 10.1166/sam.2014.2000.

8. Raghu AV, Gadaginamath GS, Mallikarjuna NN, and Aminabhavi TM (2006) Synthesis and characterization of novel polyureas based on benzimidazoline-2-one and benzimidazoline-2-thione hard segments*.* Journal of Applied Polymer Science 100(1):576-583. DOI: <https://doi.org/10.1002/app.23334>.

9. Liu H, Zhong X, Liu H, and Cheng YF (2018) Microbiologically-enhanced galvanic corrosion of the steel beneath a deposit in simulated oilfield-produced water containing Desulfotomaculum nigrificans*.* Electrochemistry Communications 90:1-5.

10. Dou W, Liu J, Cai W, Wang D, Jia R, Chen S, and Gu T (2019) Electrochemical investigation of increased carbon steel corrosion via extracellular electron transfer by a sulfate reducing bacterium under carbon source starvation*.* Corrosion Science 150:258-267.

11. Javed M, Stoddart P, and Wade S (2015) Corrosion of carbon steel by sulphate reducing bacteria: Initial attachment and the role of ferrous ions*.* Corrosion Science 93:48-57.

12. Jia R, Tan JL, Jin P, Blackwood DJ, Xu D, and Gu T (2018) Effects of biogenic H2S on the microbiologically influenced corrosion of C1018 carbon steel by sulfate reducing Desulfovibrio vulgaris biofilm*.* Corrosion Science 130:1-11. DOI: <https://doi.org/10.1016/j.corsci.2017.10.023>.

13. Dall'Agnol LT, Cordas CM, and Moura JJ (2014) Influence of respiratory substrate in carbon steel corrosion by a Sulphate Reducing Prokaryote model organism*.* Bioelectrochemistry 97:43-51.

14. Jia R, Unsal T, Xu D, Lekbach Y, and Gu T (2019) Microbiologically influenced corrosion and current mitigation strategies: a state of the art review*.* International biodeterioration & biodegradation 137:42-58.

15. AlAbbas FM, Williamson C, Bhola SM, Spear JR, Olson DL, Mishra B, and Kakpovbia AE (2013) Influence of sulfate reducing bacterial biofilm on corrosion behavior of low-alloy, high-strength steel (API-5L X80)*.* International Biodeterioration & Biodegradation 78:34-42.

16. Brug G, van den Eeden AL, Sluyters-Rehbach M, and Sluyters JH (1984) The analysis of electrode impedances complicated by the presence of a constant phase element*.* Journal of electroanalytical chemistry and interfacial electrochemistry 176(1-2):275-295.

17. Boukamp BA (2004) Electrochemical impedance spectroscopy in solid state ionics: recent advances*.* Solid state ionics 169(1-4):65-73.

18. Daniels JS and Pourmand N (2007) Label‐free impedance biosensors: Opportunities and challenges*.* Electroanalysis: An International Journal Devoted to Fundamental and Practical Aspects of Electroanalysis 19(12):1239-1257.

19. Labena A, Hegazy M, Horn H, and Müller E (2015) The biocidal effect of a novel synthesized gemini surfactant on environmental sulfidogenic bacteria: Planktonic cells and biofilms*.* Materials Science and Engineering: C 47:367-375.

20. Song W, Chen X, He C, Li X, and Liu C (2018) Microbial corrosion of 2205 duplex stainless steel in oilfield-produced water*.* Int. J. Electrochem. Sci 13:675-689.

21. Loto C (2017) Microbiological corrosion: mechanism, control and impact—a review*.* The International Journal of Advanced Manufacturing Technology 92(9):4241-4252.

22. Qu Q, Li S, Li L, Zuo L, Ran X, Qu Y, and Zhu B (2017) Adsorption and corrosion behaviour of Trichoderma harzianum for AZ31B magnesium alloy in artificial seawater*.* Corrosion Science 118:12-23.

23. Jiang D, Feng D, Jiang H, Yuan L, Yongqi Y, Xu X, and Fang W (2017) Preliminary study on an innovative, simple mast cell-based electrochemical method for detecting foodborne pathogenic bacterial quorum signaling molecules (N-acyl-homoserine-lactones)*.* Biosensors and Bioelectronics 90:436-442. DOI: <https://doi.org/10.1016/j.bios.2016.09.096>.

24. Jiang D, Liu Y, Jiang H, Rao S, Fang W, Wu M, Yuan L, and Fang W (2018) A novel screen-printed mast cell-based electrochemical sensor for detecting spoilage bacterial quorum signaling molecules (N-acyl-homoserine-lactones) in freshwater fish*.* Biosensors and Bioelectronics 102:396-402. DOI: <https://doi.org/10.1016/j.bios.2017.11.040>.

25. Jiang H, Jiang D, Shao J, and Sun X (2016) Magnetic molecularly imprinted polymer nanoparticles based electrochemical sensor for the measurement of Gram-negative bacterial quorum signaling molecules (N-acyl-homoserine-lactones)*.* Biosensors and Bioelectronics 75:411-419. DOI: <https://doi.org/10.1016/j.bios.2015.07.045>.

26. Özcan ŞM, Sesal NC, Şener MK, and Koca A (2020) An alternative strategy to detect bacterial contamination in milk and water: a newly designed electrochemical biosensor*.* European Food Research and Technology 246(6):1317-1324. DOI: 10.1007/s00217-020-03491-2.
